# Germ Cell Maintenance and Sustained Testosterone and Precursor Hormone Production in Human Prepubertal Testis Organ Culture with Tissues from Boys 7 Years+ under Conditions from Adult Testicular Tissue

**DOI:** 10.3390/cells12030415

**Published:** 2023-01-26

**Authors:** Neels Lennart Aden, Matthias Bleeke, Uwe R. Kordes, Bianka Brunne, Barbara Holstermann, Ronald Biemann, Uta Ceglarek, Armin Soave, Andrea Salzbrunn, Stefan W. Schneider, Kathrein von Kopylow

**Affiliations:** 1 Clinic and Polyclinic for Dermatology and Venerology, Andrological Section, University Medical Center Hamburg-Eppendorf, 20246 Hamburg, Germany; 2 Department of Pediatric Hematology and Oncology, University Medical Center Hamburg-Eppendorf, 20246 Hamburg, Germany; 3 Institute of Neuroanatomy, University Medical Center Hamburg-Eppendorf, 20246 Hamburg, Germany; 4 Institute of Laboratory Medicine, Clinical Chemistry and Molecular Diagnostics, University of Leipzig, 04103 Leipzig, Germany; 5 Department of Urology, University Medical Center Hamburg-Eppendorf, 20246 Hamburg, Germany

**Keywords:** prepubertal boys, fertility preservation, testis organ culture, in vitro culture, spermatogonia, spermatogonial stem cells, steroid hormone profile, liquid chromatography-tandem mass spectrometry, LC-MS/MS

## Abstract

Human prepubertal testicular tissues are rare, but organ culture conditions to develop a system for human in vitro-spermatogenesis are an essential option for fertility preservation in prepubertal boys subjected to gonadotoxic therapy. To avoid animal testing in line with the 3Rs principle, organ culture conditions initially tested on human adult testis tissue were applied to prepubertal samples (*n* = 3; patient ages 7, 9, and 12 years). Tissues were investigated by immunostaining and transmission electron microscopy (TEM), and the collected culture medium was profiled for steroid hormones by liquid chromatography-tandem mass spectrometry (LC-MS/MS). Culture conditions proved suitable for prepubertal organ culture since SSCs and germ cell proliferation could be maintained until the end of the 3-week-culture. Leydig cells (LCs) were shown to be competent for steroid hormone production. Three additional testis tissues from boys of the same age were examined for the number of germ cells and undifferentiated spermatogonia (SPG). Using TEM micrographs, eight tissues from patients aged 1.5 to 13 years were examined, with respect to the sizes of mitochondria (MT) in undifferentiated SPG and compared with those from two adult testicular tissues. Mitochondrial sizes were shown to be comparable between adults and prepubertal boys from approximately 7 years of age, which suggests the transition of SSCs from normoxic to hypoxic metabolism at about or before this time period.

## 1. Introduction

Classical oncological therapies are often gonadotoxic and cause permanent infertility due to the depletion of the spermatogonial stem cell (SSC) pool, and damage of the testicular somatic environment [1,2]. More recently, small molecule targeted therapy has also been incriminated [3,4]. On a global scale, much progress has been made in improving overall cancer survival rates, although there are regional differences due to inequities in health care access. Long-term survival (>15 years) of children/adolescents with oncological diseases, e.g., in Germany, is currently >80%, and for hemato-oncological diseases, even up to 90% [2,5,6]. Many patients reach reproductive age, which leads to a large long-term survivor cohort with a huge burden of long-term effects and fertility problems [2,7], as the primary focus in cancer patient care is often limited to direct cancer control [8].

Transplantation of hematopoietic stem cells (HSCT) is a treatment option for a variety of inborn hematologic, immunologic, and metabolic diseases. While offering curative treatment for these diseases, HSCT usually requires myeloablative conditioning with high-dose chemotherapy, particularly by using alkylating agents. The risk of infertility later in life plays an important role in the decision of parents to perform HSCT on these children [9]. Due to the accumulating complications from the respective inborn diseases, it is generally recommended to perform HSCT at prepubertal age; therefore, procedures for fertility preservation from immature germ cell tissue are much needed.

The SSCs, a subpopulation of the undifferentiated spermatogonia (SPG), are the earliest precursors of spermatids within the male adult. During testis development, SPG arise from the gonocytes by migration from the center to the periphery within the testis chords [10]. The SPG form the basis for lifelong spermatogenesis, and the unipotent SSCs, as a subpopulation of SPG, are-like other stem cells-characterized by an intrinsic self-renewal ability [11,12]. SSC homeostasis has to be balanced between self-replication for maintaining the SSC pool and further differentiation into mature spermatids starting from puberty, with the whole process regulated by the testicular microenvironment, especially the testicular somatic cells [11,13].

Fertility preservation refers to the freezing of germ cells or of gonadal tissue in the case of a given risk of infertility for the patient due to disease, medical treatment, age, or other influences [14]. Cryopreservation of sperm for fertility preservation prior to a gonadotoxic therapy, different from adult males, is not an option for prepubertal boys since no spermatids are produced before puberty. Consequently, the only possibility for fertility preservation in these cases is cryopreservation of testicular tissue, containing SSCs [2]. To date, many clinical centers worldwide cryopreserve immature testicular tissue of prepubertal males prior to a gonadotoxic treatment. However, it is unclear whether these tissues can later be used therapeutically, since all current approaches in this field are still at an experimental level, although they are constantly developing [15,16,17,18]. There have been three main fertility preservation approaches: (i) In vitro-spermatogenesis with the SSCs acting as starting cell population for the generation of spermatids in a cell culture dish, (ii) propagation of SSCs to obtain a sufficient quantity of this rare cell type for later autologous re-transplantation, (iii) autologous grafting of undissociated immature testicular tissue pieces with the aim of achieving the patient’s own in vivo-spermatogenesis [2]. A successful attempt using the third option in the study of Fayomi et al. (2019) recently resulted in the generation of a healthy rhesus monkey offspring [19]. For patients with cancer, only (i) does not carry a risk of transmitting metastatic malignant cells [2,16].

Starting from 2017, a few studies on human prepubertal testicular tissue using organ culture with the aim of propagating human SSCs and/or human in vitro-spermatogenesis have been published [20,21,22,23,24,25]. Organ culture offers the advantage of preserving normal body tissue context, and in this case, the germ cell niche, unlike conventional 2D cell culture, where the natural arrangement of the cells and the cellular interactions are lost [26,27]. Using testicular organ culture, Sato and colleagues in 2011 achieved the in vitro-production of functional murine spermatozoa from SSCs in testicular tissue pieces of the neonatal mouse testis [28]. So far, human in vitro-spermatogenesis in prepubertal human tissue containing SPG and SSCs as the only germ cells within a reliable functional approach has not yet been achieved [16,17]. Besides species-specific differences between men and mice, a major impediment in research has been the scarce availability of human prepubertal testis tissue, resulting in only a few studies within the last years. Nevertheless, due to the worldwide fertility preservation programs, awareness, and the patient’s motivation to donate a tissue piece for research, new possibilities are opened.

This study intended to evaluate organ culture conditions initially tested on adult testicular tissue, with respect to their applicability to prepubertal testis tissue. For this purpose, tissues from three boys, aged 7, 9, and 12 years, collected prior to gonadotoxic therapy, which all exhibited serum testosterone below the detection level and SPG as the most advanced germ cells, were cultured using the gas-liquid interphase method in combination with StemFlex medium without or with additive components including colony stimulating factor 1 (CSF1), gonadotropins, activin A, and nodal. Tissue analysis, before, during, and after the culture, was performed on the protein, morphological, and ultrastructural level using immunofluorescence staining, semi-thin sections, and transmission electron microscopy (TEM). The used organ culture medium was analyzed in a close-meshed rhythm (every 2nd day) with regard to its hormone steroid profile by liquid chromatography-tandem mass spectrometry (LC-MS/MS), involving the determination of testosterone, progesterone, 17-hydroxyprogesterone (17-OHP), androstenedione, and estradiol. In addition to the organ culture approach, uncultured tissues from three additional boys of the same age groups (also 7, 9, and 12 years old), also admitted for fertility preservation, were examined for their number of DEAD-box helicase 4 (DDX4)-positive germ cells and piwi like RNA-mediated gene silencing 4 (PIWIL4)-positive SPG/SSCs, reflecting the heterogeneity of the tissues in the group of patients whose testis samples are frozen in the fertility preservation programs. To demonstrate the comparability of organ culture conditions between prepubertal tissues above a certain age of patients and adult testicular specimens at the ultrastructural level, mitochondrial sizes of undifferentiated SPG were determined in eight prepubertal and two adult testicular tissues using TEM micrographs.

Here, we show that organ culture conditions developed on adult human testicular tissue are transferable to prepubertal patients 7 years and older, in regard to SPG maintenance and proliferation, which possibly can be explained by a comparability of the mitochondrial sizes in undifferentiated SPG and therefore a similar metabolism. Younger children, in contrast, displayed highly significant, larger mitochondria (MT), indicating a different mechanism of energy production. During the organ culture, Leydig cell (LC) functionality was evidenced by male steroid hormone production.

## 2. Materials and Methods

### 2.1. Patients, Testicular Biopsies and Ethical Approval

For the organ culture experiments, prepubertal testicular tissue was acquired from three prepubertal patients of the University Hospital Hamburg-Eppendorf, Department of Pediatric Hematology and Oncology, in the course of a biopsy for fertility preservation prior to a gonadotoxic therapy (high dose chemotherapy and stem cell support; see the first three patients in Table 1). Tissues from three further patients of the same age groups with the same or similar diagnoses were additionally applied for SPG and germ cell quantification.

For the measurements of the mitochondrial sizes in undifferentiated SPG, eight prepubertal and two adult testicular Epon 812-embedded specimens from patients, whose tissues had been collected as part of the diagnostic workup in the context of fertility preservation in advance of a gonadotoxic therapy or in the course of a testicular sperm extraction (TESE) [29,30], were employed (Table 2).

An informed and written consent for these studies, as well as an ethical committee approval (Approval Numbers PV7320, WF-005/13, WF-007/11; Ethics Committee Ärztekammer Hamburg) were obtained. All experiments were performed according to the Declaration of Helsinki’s ethical principles.

### 2.2. Epon Embedding, Semithin Section Technique and TEM

For diagnostic purposes, one small testis fragment (~30 mg) from each patient was immediately transferred and fixed in 5.5% glutaraldehyde in phosphate buffer (pH 7.4) for a minimum of 3 h at 4 °C, and post-fixed for 2 h at room temperature in 1% osmium tetroxide in 0.1 M sucrose phosphate buffer [29]. Embedding in Epon 812 was performed using a Leica EM TP tissue processor (Leica Biosystems, Nußloch, Germany) before semithin sections (1 µm) were cut with a diamond knife (Diatome AG, Biel, Switzerland) on a Reichert ultramicrotome (Leica Biosystems, Nußloch, Germany). Semithin sections were stained with toluidine blue and pyronine (9+1). Photos of the semithin sections were taken using an Axiovert100 microscope equipped with an Axiocam ICc3 camera (Carl Zeiss AG, Oberkochen, Germany).

For TEM, ultrathin sections (80 nm) were prepared and double-contrasted in 1% uranyl acetate for 30–60 min and Reynolds lead citrate for 3 min. Samples were photographed using a CM100 transmission electron microscope (Philips, Amsterdam, The Netherlands), equipped with a Quemesa Olympus TEM CCD camera (Olympus, Tokyo, Japan).

### 2.3. Organ Culture

Testicular biopsies were transferred intraoperatively in DMEM/F12 and quickly cooled for short-term storage in a refrigerator at 5 °C. The sample was swiftly cut in a sterile atmosphere into 11 to 23 pieces ranging from 2 mm^3^ to 4 mm^3^, depending on the size of the biopsy. One small bit of each sample was directly fixed in modified Davidson fluid (mDF) for 1.5 h at 5 °C [31], dehydrated in alcohol, and embedded in paraffin, serving as an untreated “native” reference.

The other fragments were cultured in a modified testicular explant culture system established by Sato et al. (2011) at 35 °C and 5% CO_2_ [24,28,32]. For the gel stands, 0.07 g agarose (#A9639, Sigma-Aldrich, St. Louis, MO, USA) was heated in 10 mL cell culture grade deionized water using a water bath. The completely solved agarose was further diluted using 10 mL of cell culture medium StemFlex (Gibco Life Technologies, Grand Island, NY, USA) without the supplement and 1% penicillin and streptomycin (Pen/Strept; Sigma Aldrich, St. Louis, MO, USA). One ml of the 0.35% agarose was pipetted into 24-well plates and cooled down until solidification underneath a laminar flow hood. With the backside of a sterile 1000 µL pipette tip, round gel blocks were cut out. Four blocks each were placed in a 35 mm cell culture dish with 4 inner rings (#627170, Greiner Bio GmbH, Frickenhausen, Germany). The dishes were filled with 1500 µL of StemFlex cell culture medium containing 1% Pen/Strept and further supplements [A: none; B: complex (200 ng/mL follicle-stimulating hormone (FSH), 100 ng/mL luteinizing hormone (LH), 50 ng/mL, 10^−7^ M melatonin, activin A (all from Sigma-Aldrich, St. Louis, MO, USA), 50 ng/mL nodal (R&D Systems, Minneapolis, USA), 20 ng/mL glial cell derived neurotrophic factor (GDNF) (PeproTech EC Limited, London, UK), 1 ng/mL fibroblast growth factor 2 (FGF2) (Invitrogen Gibco, Camarillo, CA, USA); C: complex + 10 ng/mL CSF1 (Sigma-Aldrich, St. Louis, MO, USA)] and soaked overnight. After placing 1–2 testicular fragments on the gel stands using tweezers, complete media changes were performed on day 1. During the rest of the cultivation period, every second day 400 µL were removed and replaced with 500 µL fresh pre-incubated medium, keeping the agarose 4/5 submerged considering evaporation. The replaced media were frozen for later diagnostics.

On days 7, 14, and 21, one to two fragments per patient and condition were retrieved quickly, keeping the time outside the incubator as short as possible. Samples were fixed in mDF as earlier described and embedded in paraffin.

### 2.4. Histological and Immunofluorescence Staining

Sections of 5 µm thickness were de-paraffinized in xylene and rehydrated through a descending alcohol series. 

For the assessment of the general histology of the cultured tissues, Mayer’s Hematoxylin was pipetted and removed after 5 min. Subsequently, the slides were submerged in tap water and blued for 3 min.

For immunofluorescence staining, heat-induced antigen retrieval was performed on using 0.05 M boric acid at pH 8.5 in a microwave oven. After washing with TBS and permeabilizing with TBS + 0.1% Tween, sections were blocked for 1 h using TBS containing 0.1% Tween and 2% normal goat serum. Afterwards, primary antibodies (Supplementary Table S1) were applied and incubated overnight at 5 °C. After washing and permeabilizing, secondary antibody incubation was performed at room temperature for 3 h in darkness (for a list of secondary antibodies used and dilutions, see Supplementary Table S2). The sections were counterstained with DAPI (1 µg/mL). The first antibody was omitted in each case for the negative controls (Figure S2). Fluorescence imaging was performed with an Axio Vert.A1 equipped with a Colibri 7 LED and an Axiocam 503 color camera (Carl Zeiss AG, Oberkochen, Germany). For confocal scanning microscopy, a Leica TCS SP5 (Leica Microsystems GmbH, Wetzlar, Germany) was utilized.

As cell type-specific markers, DDX4 (germ cells), undifferentiated embryonic cell transcription factor 1 (UTF1; SPG/SSCs), PIWIL4 (SPG/SSCs), boule homolog, RNA binding protein (BOULE, BOLL; spermatocytes (SPCs)), nuclear SRY-box transcription factor 9 (SOX9; Sertoli cells (SCs)), claudin 11 (CLDN11; SCs, blood-testis-barrier (BTB) component), alpha-smooth muscle actin (SMA; peritubular cells (PTCs)), laminin alpha 1 (LAMA1; extracellular matrix glycoprotein secreted by the PTCs), steroidogenic acute regulatory protein (STAR; LCs), cytochrome P450 family 17 subfamily A member 1 (CYP17A1; LCs), and insulin like 3 (INSL3; LCs) were applied. For determination of germ cell proliferation, Ki-67 (MKI67) was employed in combination with the germ cell-specific markers.

### 2.5. Quantification of Cells and Testicular Tubules with Respect to Cell-Type-Specific Markers

Between two and four tissue sections were examined manually per cell culture condition and week. Using the images taken on an Axio Vert.A1 microscope equipped with a Colibri 7 LED and an Axiocam 503 color camera, surface areas of the sections were measured with the Zeiss Zen 2.0 software (Carl Zeiss AG, Oberkochen, Germany). Cells were excluded if there was no DAPI counterstaining and their size and form did not fit the corresponding cell type, e.g., SPG, and they did not show the typical staining for the respective marker. For LAMA1 quantification, tubules were counted as LAMA1+, if at least 3/4 of the basement membrane showed the cell-specific marker expression.

### 2.6. Terminal Deoxynucleotidyl Transferase-Mediated dUTP Nick-End Labeling (TUNEL)

For the detection of dead cells/areas in the tissue, TUNEL staining was performed using the DeadEndTM Fluorometric TUNEL System (#G3250, Promega, Madison, WI, USA). For the protocol, rehydrated sections as for immunofluorescence staining, were used. These were further processed according to the manufacturer’s protocol. Cells were counterstained with DAPI (1 µg/mL; Thermo Scientific, Rockford, IL, USA) and mounted with glass coverslips. Negative controls were performed without the rTdT enzyme; positive controls were treated with 1500 U/mL DNase1. Imaging was done with an Axio Vert.A1 equipped with a Colibri 7 LED and an Axiocam 503 color camera. For quantification, the channel that represented TUNEL was separated, and the area was estimated using an intensity threshold in ImageJ (U.S. National Institutes of Health, Bethesda, MD, USA). Subsequently, the area of the whole section was measured, and the percentage of the area containing cells with fragmented DNA was calculated. 

### 2.7. Enzyme-Linked Immunosorbent Assay (ELISA)

Initially, ELISA assays were performed to determine testosterone levels in organ culture supernatants using the Testosterone Human ELISA Kit ab174569 (Abcam, Cambridge, UK). Assays were carried out according to the manufacturer’s protocol. Briefly, a calibrator, samples, and positive controls were dispensed into the wells. HRP enzyme conjugate was added, mixed thoroughly, and incubated for 60 min at room temperature on a plate shaker. After striking out the contents of the wells and washing, TMB substrate solution was added, and the plate was incubated for 15 min. The reaction was stopped with a stop solution, and absorbance of each well was determined at 450 ± 10 nm using an Anthos HT3 microplate reader (Biochrom, Cambridge, UK). The results have been calculated automatically via a 4 PL (4 Parameter Logistics) curve fit. Samples with a concentration higher than the range of the calibrator have been diluted. Serum from a 25-year-old healthy male was used as a control.

Due to an unsuitable standard concentration series for the level of testosterone concentration to be determined in the organ culture supernatant of the prepubertal samples, and thus a pre-programmed measurement inaccuracy using the ELISA assay, samples from the patients with measured absorbance values were referred to liquid chromatography-tandem mass spectrometry (LC-MS/MS) (see next section).

### 2.8. Determination of Steroid Hormones by LC-MS/MS

Steroid hormones in organ culture supernatants were determined at the Institute of Laboratory Medicine, Clinical Chemistry and Molecular Diagnostics, University of Leipzig (Leipzig, Germany). A 100 µL of supernatants were used for the simultaneous quantification of 17-OHP, aldosterone, androstenedione, cortisol, cortisone, dehydroepiandrosterone sulfate (DHEAS), estradiol, progesterone, and testosterone using online solid phase extraction (SPE) LC–MS/MS as previously described [33]. Lower limits of quantitation ranged from 47 pmol/L (estradiol) to 69 pmol/L (aldosterone), 0.1 nmol/L (testosterone, DHEAS), 0.2 nmol/L (androstenedione, progesterone, cortisol), and 0.3 nmol/L (17-OHP). General accuracy was 87–109%, with a between-run imprecision ≤ 10%. 

### 2.9. Measurement of Mitochondrial Sizes in Undifferentiated SPG

For these investigations, TEM micrographs were used. Photos were imported into the Zeiss Axiovision software (Carl Zeiss AG, Oberkochen, Germany). For correct scaling, the length of the scale bar in pixels was manually measured. Subsequently, the surface areas of the MT were manually determined using the spline function. To exclude a bias due to the differential weighting of cells with many MT and cells with only a few MT, the mitochondrial sizes of the undifferentiated SPG were surveyed in a cell-independent manner. In total, 1023 MT of prepubertal SPG and 428 MT of adult SPG were determined. Statistics were calculated using a pairwise *t*-test with Bonferroni multiple-comparison correction.

### 2.10. Statistical Testing, Linear Regression and Spearman Correlation Analysis

Prior to statistical analysis, the samples were tested for normal distribution of the values by means of the Kolmogorov-Smirnov test [34]. Statistical tests were conducted using the Kruskal Wallis test (germ cell quantification) and a pairwise *t*-test with Bonferroni multiple-comparison correction (measurement of the mitochondrial sizes) [35,36]. To investigate linear and nonlinear dependencies between the steroid hormone levels, linear regression analysis and Pearson correlation analysis were carried out [36,37].

## 3. Results

### 3.1. Status and Viability of the Tissues Prior and during Organ Culture

In order to study if organ culture conditions previously tested on adult human testicular tissue are suitable to establish a model for human prepubertal ex vivo-spermatogenesis, testis tissue from three prepubertal boys was cultured under three different organ culture conditions using StemFlex medium containing 1% Pen/Strept. In condition A, the medium without further additives was utilized, whereas conditions B and C were supplemented, B with FSH, LH, melatonin, activin A, nodal, GDNF, and FGF2. For condition C, CSF1 was additionally added. Initially, a culture period of three weeks was planned for each tissue. 

To assess the status of the tissue pieces during organ culture, at the respective time points at which a portion of the culture was completed (1 week, 2 weeks, 3 weeks), mDF-fixed tissue was stained with Mayer’s Hematoxylin. This revealed in the case of the 9-year-old sickle cell disease (SCD) patient that, under culture, a good preservation of the tissue could not be achieved due to the presence of many fibrous areas and the depletion of germ cells (Figure S3). By means of DDX4 staining per time point and condition, this finding could be confirmed. Hence, this cultivation approach was discontinued after only two weeks (more details in Section 3.4).

**Figure 1:**
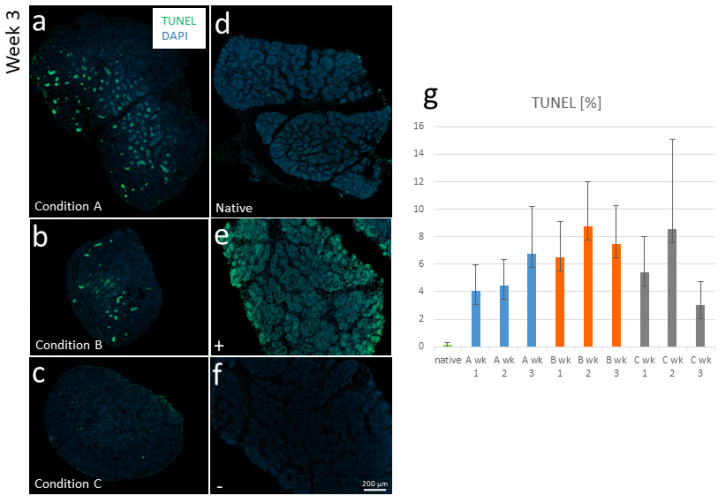
TUNEL staining for assessment of tissue preservation in prepubertal testicular tissue before and during organ culture. (**a**–**c**) Representative images from the culture with the tissue from the 7- and 12-year-old patients after 3 weeks (wk) under conditions A (**a**), B, (**b**) and C (**c**). (**d**) Status of native tissue. (**e**) Positive, and (**f**) negative control. Scale bars: 200 µm. (**g**) Results from the quantification of TUNEL+ areas for determination of tissue preservation before (native) and during organ culture under the 3 culture conditions used (before culture, green bar; condition A, blue bars; condition B, orange bars; condition C, grey bars). Values shown as mean ± standard deviation from *n* = 2 biological replicates with 1–2 technical replicates from each patient per time point and condition. From each sample, 2 independent tissue sections were analyzed.

To further assess the status of the two other cultivated tissues in terms of dead areas due to the existence of cells whose DNA is fragmented, TUNEL staining was performed (Figure 1a–f). This revealed a proportion of approximately 0.04–0.3% of TUNEL+ areas in the uncultured (native) tissues and 3–9% of TUNEL+ areas in the cultured tissues (see Figure 1g).

### 3.2. Organ Culture Conditions Promote Maturation of PTCs

**Figure 2:**
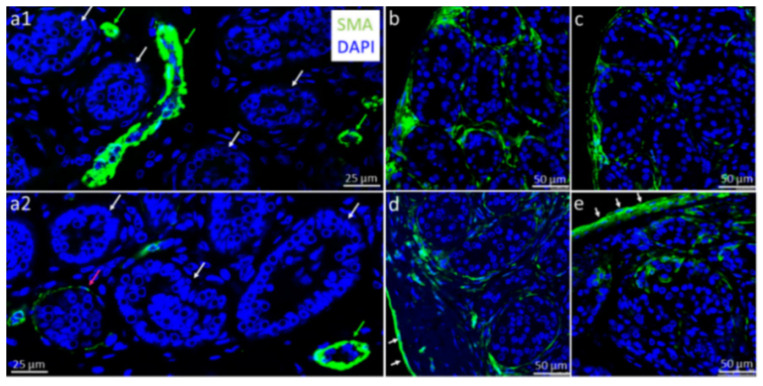
SMA protein expression in the prepubertal testis tissue before and during organ culture. (**a**) Native tissue (before culture). The green arrows in (**a1**,**a2**) mark strong SMA expression in blood vessel walls; the pink arrow in a2 marks one seminiferous tubule showing SMA expression in the basement membrane; and the white arrows in a1 and a2 highlight seminiferous tubules without SMA expression in the basement membrane. (**b**) Organ culture after 1 week under condition A; and (**c**) C; (**d**) culture after 2 weeks under condition B. Arrows mark the layer of SMA-expressing PTCs surrounding the cultured testicular tissue. (**e**) Organ culture after 3 weeks under condition B. Arrows mark the layer of SMA-expressing PTCs surrounding the cultured testicular tissue. (**b**,**c**,**e**) Testis tissue of the 7 years old, and (**a1**,**a2**,**d**) the 12 years old patient. Scale bars: 25 µm (**a1**,**a2**) and 50 µm (**b**–**e**).

In the native testis tissue of the 7-year- and 12-year-old patients before culture, the alpha-SMA protein expression was predominantly seen in the interstitium, especially in the blood vessel walls (Figure 2(a1), green arrows), whereas the PTCs in the basement membrane of the seminiferous tubules showed hardly any expression (Figure 2(a1,a2), white arrows). During organ culture, a maturation of the PTCs occurred, as could be observed by the PTCs starting to express alpha-SMA [38,39], chiefly in the peripheral areas (Figure 2b–e). Similarly, as in the marmoset cultures of Sharma et al. (2022) [39], we observed encapsulation of the organ culture pieces with a layer of cells expressing alpha-SMA over the cultivation period, especially in the tissue of the 7-year-old patient (Figure 2e). In the tissue of the 12-year-old boy, this was much less pronounced (Figure 2d). Regarding alpha-SMA protein expression and PTC differentiation, no major obvious differences at the correspondingly same time points could be observed for the three culture conditions used.

### 3.3. LAMA1 Expression of PTCs Is Maintained until the End of Culture; Hormones and Growth Factors Promote a Better Maintenance of LAMA1 Expression

LAMA1 protein represents a specific component of the innermost part of the seminiferous tubule basement membrane [24]. According to Kurek and colleagues, LAMA1 expression must be maintained during the culture period as an essential prerequisite for the preservation of SPG located at the basement membrane when performing testicular organ culture.

**Figure 3:**
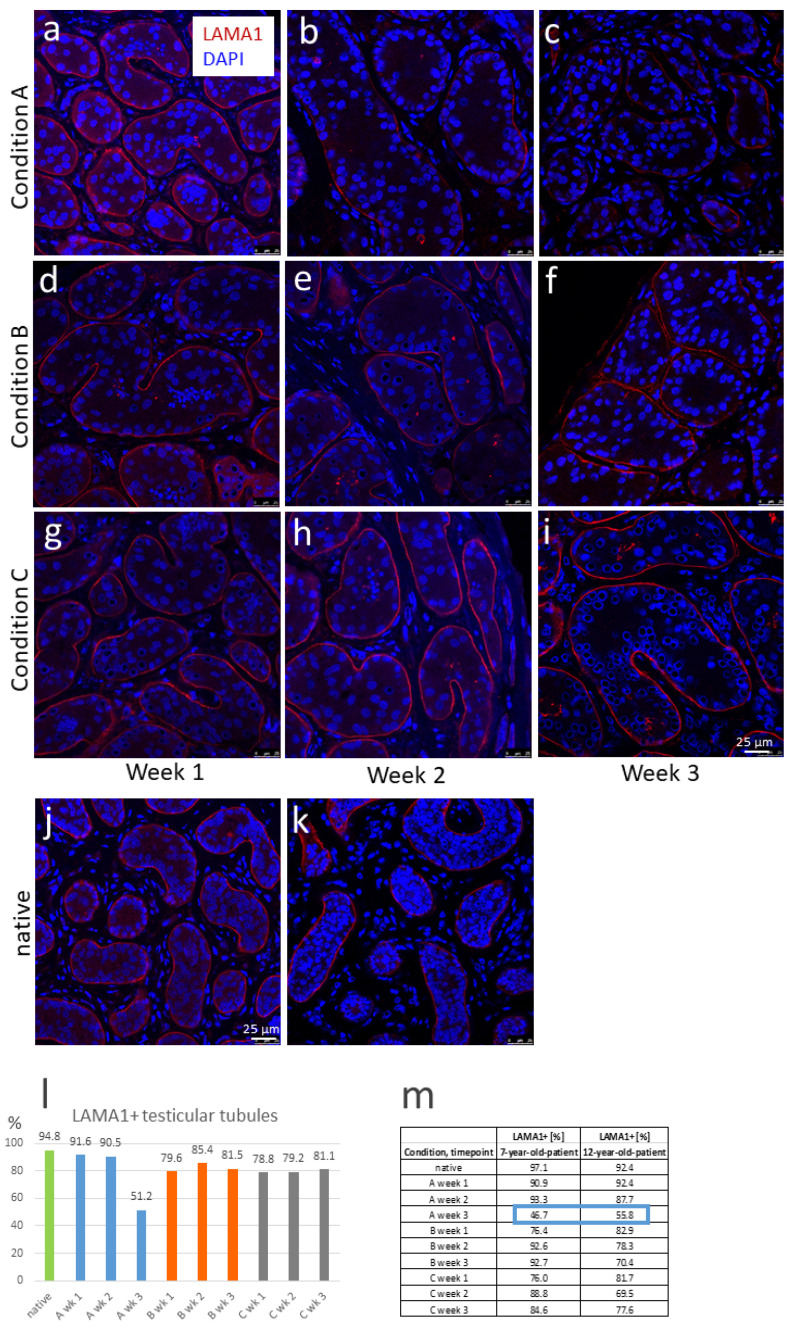
LAMA1 protein expression by immunofluorescence staining in the testis tissue of the 7-year-old and the 12-year-old patient. (**a**–**i**) Representative images of immuno-stained cross-sections from the organ culture under conditions A, B, and C after 1, 2, and 3 weeks. (**j**,**k**) Immuno-stained cross sections of the native tissue of the patients; (**a**,**d**,**e**,**g**,**h**–**j**) testis tissue of the 7-year-old patient, and (**b**,**c**,**f**,**k**) the 12-year-old patient. Scale bars: 25 µm. (**l**) Quantification of LAMA1+ seminiferous tubules in the native tissue (before culture; green bars) and after 1, 2 and 3 weeks (wk) of culture under conditions A (blue bars), B (orange bars,) and C (grey bars) in the tissue sections of the 7- and 12-year-old patients, with values shown as a mean from *n* = 2 biological replicates with 1–2 technical replicates from each patient per time point and condition. (**m**) Individual patient values, with the blue box indicating reduced LAMA1 expression under condition A in week 3.

In our study, LAMA1 protein expression in native and cultured tissue specimens from the 7-year-old and the 12-year-old patients was examined by immunofluorescence staining. These investigations showed sustained LAMA1 expression until the end of the culture period of 3 weeks, indicating the preservation of the structural integrity of the cultured tissue fragments (Figure 3a–i) compared to the native tissue pieces (status before culture; Figure 3j,k). To determine the percentage of this structural maintenance of cultured tissues per time point and culture condition, quantification of LAMA1+ seminiferous tubules was conducted. This did not reveal 100% LAMA1+ tubules in the native tissue of the patients, but slightly lower values (Figure 3l green bar, and Figure 3m). During the culture time, LAMA1 expression remained close to the initial level in condition A (blue bars) until the end of week 2, whereas only about half of the LAMA1 expression level was maintained at the end of week 3 (Figure 3l, right blue bar, and Figure 3m, blue box; see also Figure 3c). In contrast, the conditions B (left orange bar in Figure 3l,m) and C (left grey bar) each provided a worse value with respect to LAMA1 expression after 1 week of culture compared to condition A, with 15% fewer LAMA1+ tubules for B and C in the case of the 7-year-old boy vs. 10% fewer LAMA1+ tubules for B and C in the case of the 12-year-old boy (Figure 3m). Afterwards, slight changes were detected in both patients: While the 7-year-old patient showed a slight “improvement” in the values for both conditions, the 12-year-old patient showed an opposite trend with a slight “deterioration” for both conditions. However, no such strong decrease in LAMA1+ tubules could be detected in weeks 2 and 3 as in condition A in week 3. 

The strong decrease of LAMA1 expression in week 3 in condition A could indicate that this condition (without hormones and growth factors) provides worse conditions for the maintenance of LAMA1 expression in the PTCs over a longer organ culture time. However, this evidence cannot be proven here due to the small number of samples in our study.

### 3.4. Maintenance of Germ Cells (DDX4+) and SPG (PIWIL4+) over the Cultivation Period; 9-Year-Old SCD Patient as an Exception

To investigate the maintenance and development of the germ cells in the tissues during the 3-week-culture period compared to the presence of the cells in the native tissue prior to the culture, DDX4, UTF1, PIWIL4, and BOULE were employed as markers for immunofluorescence staining. While DDX4 is considered a pan-germ cell marker [40], UTF1 and PIWIL4 are expressed in SPG and SSCs [41,42]. BOULE has been reported to represent a marker for SPCs [43].

First, all three patient tissues used for the organ culture were stained for DDX4. To simultaneously visualize the proliferating germ cells, this was performed in a costaining with the proliferation marker Ki-67. Since the preservation of DDX4+ cells in the tissue of the 9-year-old SCD patient was very poor (see also Section 3.1.), the culture was stopped after 2 weeks. In this context, it is interesting to note that the native tissue of this patient completely lacked proliferating germ cells (cell type DDX4+/Ki-67+). Furthermore, in the case of this patient it was remarkable that the germ cells of the type DDX4+/Ki-67- were not evenly distributed in the tissue, but clustered in single seminiferous tubules. 

**Figure 4:**
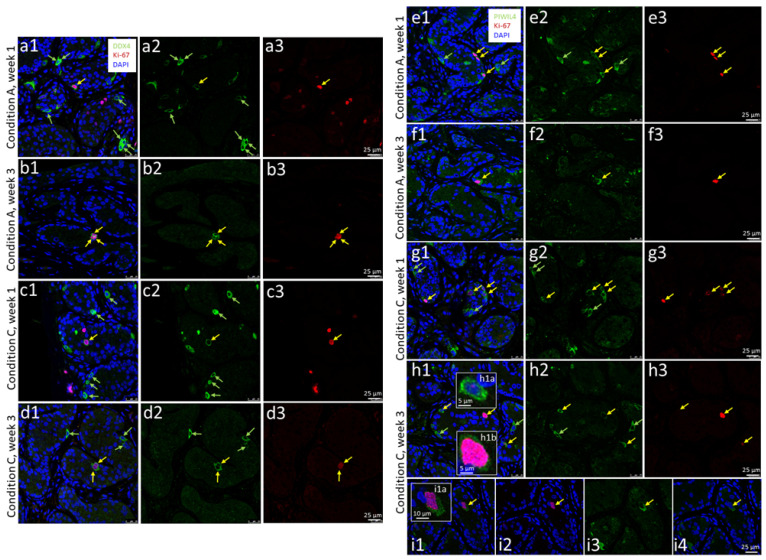
DDX4 and PIWIL4 protein expression in prepubertal testis organ culture. (**a**–**d**) Confocal images of DDX4/Ki-67 double staining of cross sections, exemplarily shown on the tissue of the 12-year-old patient. (**a**) Culture condition A after 1 week, and (**b**) after 3 weeks. (**c**) Condition C after 1 week, and (**d**) after 3 weeks. (**a1**–**d1**) Overlay images from DAPI (blue), DDX4 (green), and Ki-67 (red) staining. (**a2**–**d2**) DDX4 staining, and (**a3**–**d3**) Ki-67 staining. Yellow arrows indicate proliferating (DDX4+/Ki-67+) and green arrows indicate non-proliferating (DDX4+/Ki-67-) germ cells. Scale bars: 25 µm. (**e**–**i**) PIWIL4/Ki-67 double staining, exemplarily shown on the tissue of the 12-year-old patient. (**e**) Culture condition A after 1 week, and (**f**) 3 weeks. (**g**) Condition C after 1 week, and (**h**,**i**) 3 weeks. (**e1**–**i1**) Overlay images from DAPI (blue), PIWIL4 (green), and Ki-67 (red) staining. (**e2**–**h2**,**i3**) PIWIL4 staining, and (**e3**–**h3**) Ki-67 staining. (**i2**) DAPI, Ki-67 overlay, and (**i4**) DAPI, PIWIL4 overlay. (**h1a**,**h1b**) Magnified details from h1, and (**i1a**) from i1 present PIWIL4+/Ki-67+ cells with (**h1a**) exclusive Ki-67 nucleolus staining, (**h1b**) complete Ki-67 nuclear labeling, and (**i1a**) a mitotic figure exhibiting Ki-67 labeled chromosomes. Yellow arrows indicate spermatogonia (SPG)/spermatogonial stem cells (SSCs) with simultaneous PIWIL4 expression (PIWIL4+/Ki-67+), green arrows indicate PIWIL4+ SPG without Ki-67 expression (PIWIL4+/Ki-67-). Scale bars: 25 µm (**e1**–**i4**), 5 µm (**h1a**,**h1b**), 10 µm (**i1a**).

Qualitative analysis regarding the presence of DDX4+ cells in the cultured samples from the two other patients revealed that both proliferating (Figure 4a–d, yellow arrows) and non-proliferating DDX4-positive cells (Figure 4a–d, light green arrows) have been preserved in the tissues of the 7-year-old and the 12-year-old patient in organ culture under all three organ culture conditions used. The co-expression of DDX4 with the marker Ki-67 in the cultured tissues shows the sustained proliferative capacity of the germ cells under the selected conditions until the end of the culture period.

For the detection of undifferentiated SPG, including the SSCs, UTF1 was initially planned to be applied, normally representing a highly specific and robust protein marker for this cell type [42,44,45,46]. When we noticed that this marker, especially in the cultured prepubertal samples, also caused staining of the SCs, although much weaker (Figure S8), we changed our strategy and replaced UTF1 with PIWIL4. At the mRNA level, PIWIL4 represents the earliest SSC marker, as demonstrated by single-cell sequencing studies [42,47,48,49]. However, since we had previously found the PIWIL4 protein as being partially coexpressed with the proliferation marker Ki-67, the PIWIL4 immunostaining was also performed as a double staining with Ki-67.

These investigations revealed that both PIWIL4+/Ki-67+ (Figure 4e–i, yellow arrows) and PIWIL4+/Ki-67- cells (Figure 4e–i, light green arrows) could be preserved in the tissues of both patients until the end of the culture period.

To compare the three organ culture conditions in terms of their influence on germ cell and SPG/SSC maintenance, we performed quantification of the cells in the tissues double stained for DDX4/Ki-67 and PIWIL4/Ki-67. 

**Figure 5:**
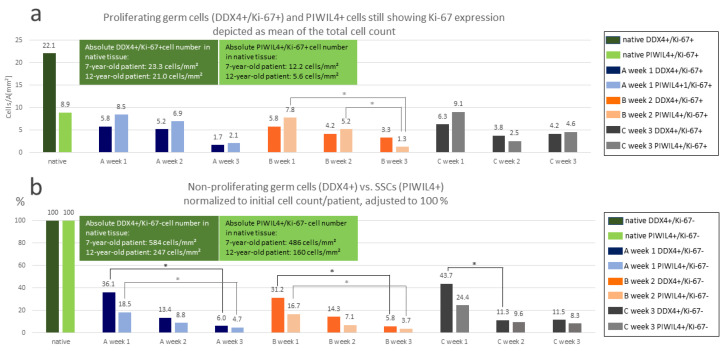
Quantification of germ cells and spermatogonia (SPG)/spermatogonial stem cells (SSCs) in the uncultured (native; green) and cultured tissues from the 7- and the 12-year-old patient under conditions A (blue), B (orange), and C (grey bars) based on immunofluorescence double staining for DDX4/Ki-67 and PIWIL4/Ki-67. (**a**) Proliferating germ cells (DDX4+/Ki-67+) and PIWIL4+ cells with Ki-67 protein expression (PIWIL4+/Ki-67+). Bars represent the absolute number of each cell population per area in mm^2^, presented as mean with * *p* ≤ 0.01. (**b**) Non-proliferating (DDX4+/Ki-67-) and PIWIL4+/Ki-67- SPG/SSCs as normalized to the initial cell count per cell population and per patient (green boxes), adjusted to 100% and shown as mean of the percentage of the maintained cells with * *p* ≤ 0.01.

Due to the poor condition of the cultured tissue from the 9-year-old SCD patient, in this case only the DDX4/Ki-67 staining was carried out and quantified (Figure S9). As the tissue showed only very few to no cells, a very uneven cellular distribution, and large differences even between tissue pieces cultured under the same conditions, these samples were excluded from the comparison of the three organ culture conditions. For this purpose, both the DDX4/Ki-67- and PIWIL4/Ki-67-stained tissues of the 7- and 12-year-old patients were used. Results are shown in Figure 5. Please note, that the DDX4+/Ki-67+ and PIWIL4+/Ki-67+ cells are shown in their absolute numbers on the basis of a comparable cell count between the two patients (Figure 5a, green boxes), whereas as a result of the differences in the initial DDX4+ and PIWIL4+ cell numbers of the two patients, a normalization had to be conducted in which the initial cell numbers in both cases were set to 100% (Figure 5b). 

**Figure 6:**
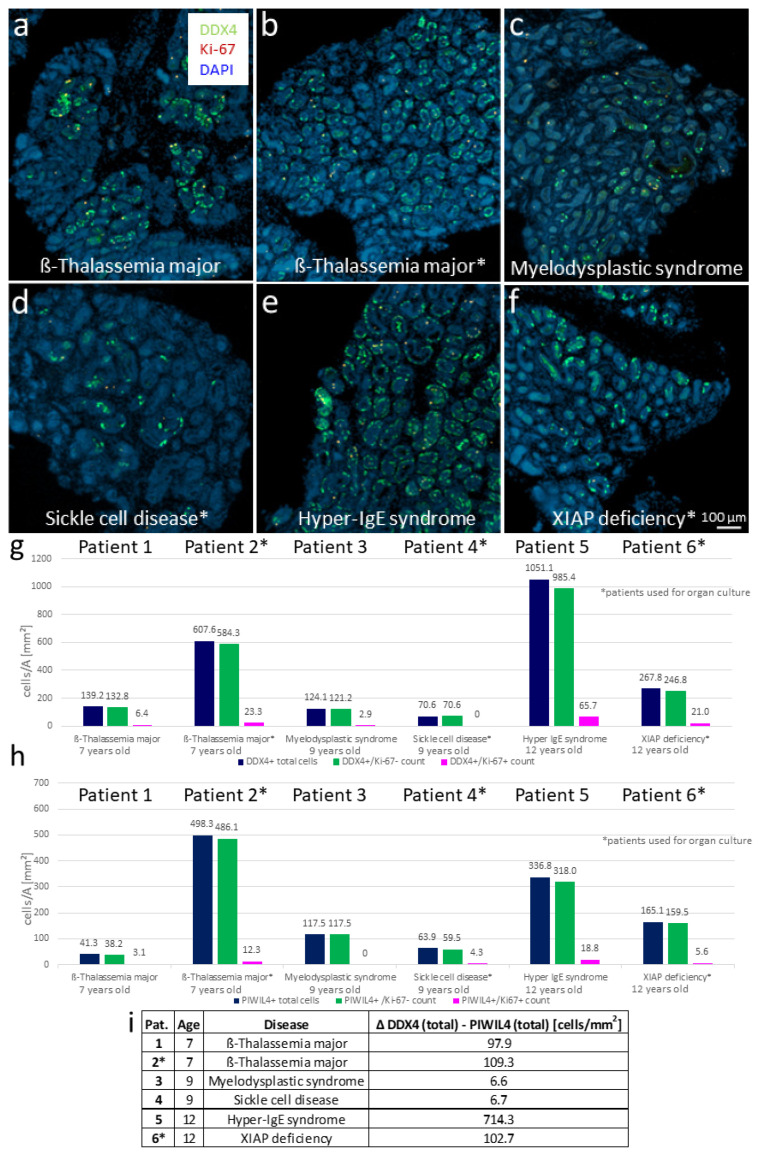
Heterogeneity in the germ cell and the spermatogonia (SPG)/spermatogonial stem cell (SSC) count in prepubertal testis tissue of patients with different ages and pathologies demonstrated by means of DDX4- and PIWIL4-double staining with Ki-67. (**a**–**f**) Immunofluorescence DDX4/Ki-67 double staining; overview images; DDX4, green; Ki-67, red; DAPI, blue. Scale bar: 100 µm. (**a**,**b**) Patient age 7 years, (**c**,**d**) 9 years, and (**e**,**f**) 12 years. (**g**) Quantification of the samples shown in (**a**–**f**) with the total germ cell count (DDX4+) in dark blue, non-proliferating germ cells (DDX4+/Ki-67-) in green, and proliferating germ cells (DDX4+/Ki-67+) in pink. Cell number shown as cells/mm^2^. (**h**) Quantification of the samples from the same patients with the total count of SPG/SSCs (PIWIL4+; dark blue), PIWIL4+/Ki-67- SPG/SSCs (green), and PIWIL4+ cells still showing Ki-67 expression (PIWIL4+/Ki-67+; pink). (**i**) Difference between the total count of DDX4+ cells and PIWIL+ cells. * Tissues from these samples were also utilized for organ culture.

The overall quantification revealed a decreasing trend with respect to all four cell populations (DDX4+/Ki-67+, PIWIL4+/KI-67+, DDX4+/Ki-67-, PIWIL4+/Ki-67-) starting from the native tissue up to cultivation week 3 with partly significant and partly non-significant statistical values (Figure 5a,b). In the case of the DDX4+/Ki-67+ cells in cultivation week 1, there was an approximately equal percentage of cells found (5.8 vs. 5.8 vs. 6.3 cells/A [mm^2^]) in comparison of the three culture conditions (Figure 6a). Roughly the same was observed in cultivation week 1 for the PIWIL4+/Ki-67+ cells (8.5 vs. 7.8 vs. 9.1 cells/A [mm^2^]) (Figure 5a), the DDX4+/Ki-67- cells (36.1 vs. 31.2 vs. 43.7% cells), and the PIWIL4+/Ki-67- cells (18.5 vs. 16.7 vs. 24.4% cells) (Figure 5b). It is also noteworthy that in each of these cases, a slightly better value for culture condition C could be recorded (the last of the 3 values in each of the 4 brackets, see above). Additionally, at week 3, based on evaluation of all cell populations (shown in Figure 6a,b), condition C seemed to turn out to be the best for the preservation of DDX4+ germ cells and PIWIL4+ SPG. However, statistical testing indicated no significant differences between the three culture conditions used.

With regard to the DDX4+ and the PIWIL4+ cell quantities in the native tissues, we observed large differences between the three patients. In the case of the non-proliferating DDX4+ germ cells (DDX4+/Ki-67-), the 9-year-old patient with SCD displayed the lowest number with 71 cells/mm^2^ (Figure 6g), the 7-year-old patient with 584 cells/mm^2^ (Figure 5b, dark green box, and Figure 6g) the highest, and the 12-year-old XIAP deficiency patient with 247 cells/mm^2^ an intermediate cell count. Similar differences in the cell numbers were detected for the PIWIL4+/Ki-67- cells in the tissues of the 7-year-old patient and the 12-year-old XIAP deficiency patient (486 cells/mm^2^ vs. 160 cells/mm^2^, respectively; Figure 5b, light green box, and Figure 3h).

### 3.5. Evaluation of Further Uncultured Prepubertal Tissues with Regard to the Proportion of DDX4+ and PIWIL4+ Germ Cells Displays the Great Heterogeneity of the Prepubertal Tissues Despite Same Age Groups and Partly Similar Diseases

To further address the fact of the heterogeneity of the prepubertal tissues regarding the germ and spermatogonial cell count, tissues from three additional patients were examined for their number of DDX4-positive and PIWIL4-positive cells. Overview photos of the DDX4/Ki-67 double-stained tissues from all six patients are depicted in Figure 6a–f. In the quantification, a distinction was again made between the DDX4+/Ki-67- cells (Figure 6g, green bars) and the DDX4+/Ki-67+ cells (Figure 6g, pink bars), or the PIWIL4+/Ki-67- cells (Figure 6h, green bars) and the PIWIL4+/Ki-67+ cells (Figure 6h, pink bars). The total number of the DDX4+ or PIWIL4+ cells is displayed by the dark blue bars in Figure 6g,h. This investigation confirmed the heterogeneity of the prepubertal tissues from the patients of different ages and pathologies. With respect to the quantification of the DDX4+ cells, the results were as follows: The 9-year-old patient with SCD remains the one with the lowest number, even when all six patients are considered (Figure 6g, patient 4, and overview photo in Figure 6d). He is followed by the 9-year-old patient with myelodysplastic syndrome (MDS) (Figure 6g, patient 3, and Figure 6c) and the 7-year-old patient with ß-thalassemia major, whose tissue was not used for the organ culture (Figure 6g, patient 1, and Figure 6a). The second 7-year-old ß-thalassemia patient (tissue was applied for the organ culture) displayed a markedly higher proportion of DDX4+ cells (Figure 6g, patient 2, and Figure 6b) compared to the ß-thalassemia major patient mentioned earlier. The 12-year-old XIAP-deficient patient exhibited approximately twice as many DDX4+ cells (Figure 6g, patient 6, and Figure 6f) as the 9-year-old MDS patient and the 7-year-old ß-thalassemia major patient with the lower value. The highest amount of DDX4+ cells was shown by the 12-year-old patient suffering from hyper-IgE syndrome (Figure 6g, patient 5, and Figure 6e).

In terms of the number of PIWIL4+ SPG/SSCs, the ranking from the lowest to the highest cell percentage is somewhat different from that for the DDX4+ cells (see Figure 6h). The tissue with the lowest cell count was that of the 7-year-old ß-thalassemia major patient (not used for organ culture) (Figure 6h, patient 1), followed by the 9-year-old SCD patient (patient 4), the 9-year-old MDS patient (patient 3), and the 12-year-old patient with XIAP deficiency (Figure 6h, patient 6). The 12-year-old hyper-IgE syndrome patient (patient 5) presented approximately twice as many PIWIL4+ cells as the XIAP deficiency patient (patient 6), while the 7-year-old ß-thalassemia major patient whose tissue was applied for the organ culture had the highest PIWIL4+ cell count (Figure 6h, patient 2). 

At first glance and with respect to the ranking, there seems to be no direct dependence between the number of DDX4-positive and PIWIL4-positive cells in the native tissues of the patients studied here. Nevertheless, upon closer inspection, an interesting pattern appears: Firstly, in the case of the two 7-year-old patients with ß-thalassemia major, the proportion of the total number of DDX4-positive cells was approximately 100 cells/mm^2^ higher than the proportion of the total number of PIWIL4-positive cells (see 1st and 2nd blue bars in Figure 6g vs. Figure 6h, and 1st vs. 2nd line of the calculated values in Figure 6i). Secondly, in the two 9-year-old patients, a consistent picture was noticeable, contrary to a fairly equal number of DDX4-positive cells compared to the PIWIL4-positive cells (3rd and 4th blue bars in Figure 6g vs. Figure 6h, and 3rd vs. 4th line of the calculated values in Figure 6i). In the case of the two 12-year-old patients, no consistent pattern emerged (5th and 6th blue bars in Figure 6g vs. Figure 6h, and 5th vs. 6th line of the calculated values in Figure 6i), but, like the 7-year-old boys, the 12-year-old XIAP-deficient patient showed a difference of approximately 100 cells/mm^2^ between the total number of DDX4-positive and PIWIL4-positive cells. Only in the case of the 12-year-old boy with the hyper-IgE syndrome, a much larger difference between the total proportions of the DDX4+ and PIWIL4+ cells (714 cells/mm^2^) was displayed. These findings are discussed in Section 4.3.

### 3.6. Presence of the SPC Marker BOULE in the Cultured versus the Native Tissue

**Figure 7:**
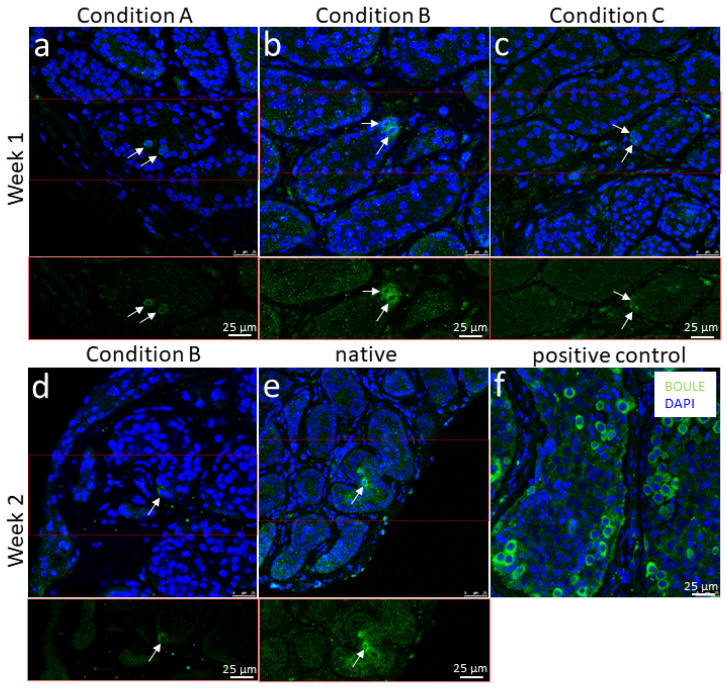
Immunofluorescence staining for the spermatocyte (SPC) marker BOULE in the prepubertal specimen during testis organ culture. (**a**) Culture condition A, and (**b**) condition B after 1 week, tissue of the 12-year-old patient. (**c**) Condition C after 1 week, tissue of the 7-year-old patient. (**d**) Culture condition B after 2 weeks, tissue of the 12-year-old patient. (**e**) Native sample (before culture) from the 7-year-old patient. (**f**) Positive control, adult testicular tissue. (**a**–**e**) Top rows show overlay images with DAPI, bottom rows show the cut-outs of relevant areas from the single Alexa 488 channel. Arrows indicate BOULE+ SPCs. Scale bars: 25 µm.

In both experiments, few positive BOULE signals were detectable after the first week and even in one sample after day 14 (Figure 7). Interestingly, in the case of the 12-year-old boy with XIAP deficiency, no BOULE+ cells were found in the untreated tissue piece (before culture, see Table 1 in Materials and Methods). However, in conditions A and B, few signals were detected after week 1 (Figure 7a,b), and even after week 2 in condition B (Figure 7d). In the native sample of the 7-year-old with ß-thalassemia major, there were 1-2 single BOULE+ cells (Figure 7e). Signals were also found in conditions A (not shown) and C (Figure 7c) after the first week. Even though the first case contained no positive signals in the specimen, we suggest that the signals in the samples after week one were already preexistent. Due to the fact that these BOULE signals were so rarely spread across the tissue, it is more likely that there may have been positive cells within the cultured pieces since the beginning and not in the sample that was picked as a reference for day 0. After 3 weeks of culture, no BOULE+ cells could be recorded. A tissue sample from the adult testis acted as a positive control here (Figure 7f). None of the negative controls showed a positive BOULE or BOULE-like signal (see Figure S2).

### 3.7. Characterization of SCs by Immunofluorescence Staining and TEM: TEM Shows SC Maturation Trends as SC Nuclei Become More Lobulated after 3 Weeks of Cultivation

In the native tissues of both patients, nuclear SOX9 expression as well as cytoplasmic expression of the BTB component CLDN11 was present, signaling a certain and approximately concordant maturation status of the SCs in the two tissues cultured over three weeks (Figures S4j,k and S5a,b,e,f). During the organ culture, expression of both proteins was maintained until the end of the cultivation period under all three conditions used (Figures S4a–l and S5c,d,g,h). Beside SOX9 expression, SC nuclei showed mild UTF1 expression (Figure S8), although this was weaker than the UTF1 expression usually found in the SPG. UTF1 expression in the SCs of the prepubertal tissues may result from the fact that the cells at this stage of development have not yet reached their full maturation status despite showing nuclear SOX9 expression. However, in our previous studies on the human adult testis, we had also observed weak UTF1 protein expression in some adult SC nuclei [50].

**Figure 8:**
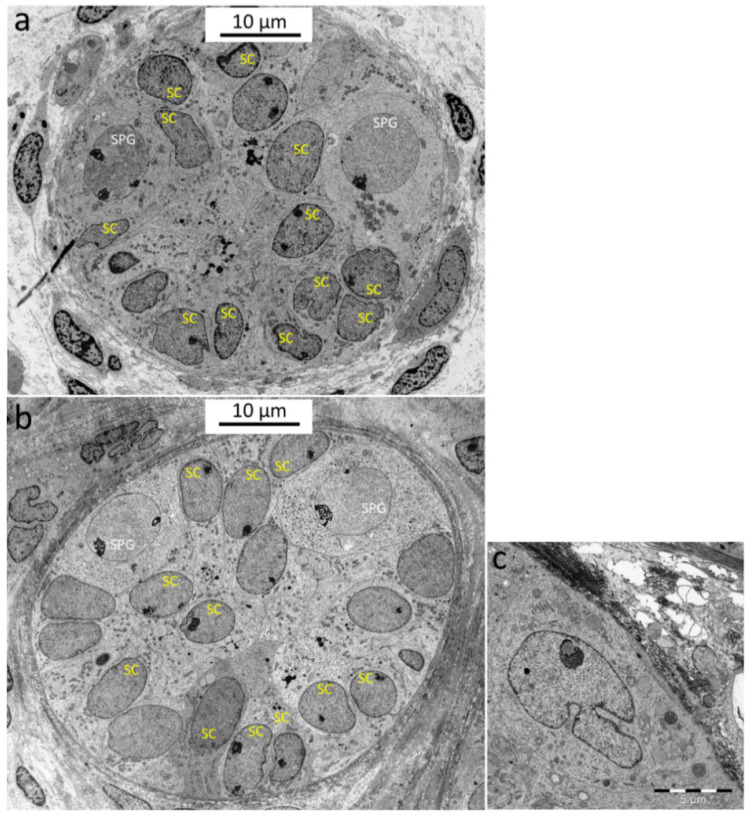
Visualization of Sertoli cells (SCs) by transmission electron microscopy (TEM). (**a**) Native tissue of the 7 years old patient, and (**b**) the 12 years old patient. SCs are marked in yellow and spermatogonia (SPG) in white. Scale bars: 10 µm. (**c**) SC with a lobulated nucleus and prominent nucleolus in the 3-week-cultured tissue (condition B). Scale bar 5 µm (**c**).

To further elucidate the maturation status of the prepubertal SCs at the ultrastructural level, we investigated the native tissues of the 7- and 12-year-old boys by TEM. Indeed, it could be seen that the SC nuclei often appeared in a more immature form, namely, more rounded and less lobulated compared to adult SCs. They also presented a less prominent nucleolus (Figure 8a,b). In contrast, this cell type appeared to undergo a maturation process during organ culture, as the nuclei tend to become more lobulated after 3 weeks of cultivation (Figure 8c). Nevertheless, due to the rarity of the cultured tissue, this finding could only be obtained for the cultures under conditions B and C and the 12-year-old patient, which were the only samples that could be examined.

### 3.8. LC Functionality Evidenced by Testosterone and Precursor Hormone Secretion

**Figure 9:**
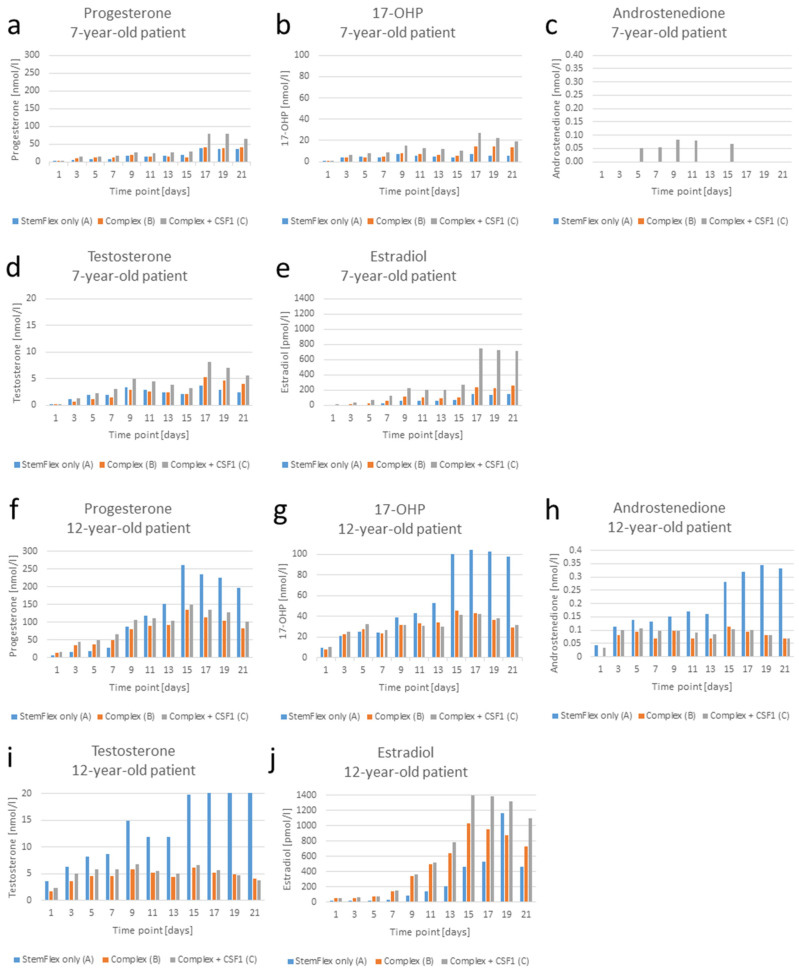
Steroid hormone profile in the organ culture supernatant over a period of 21 days, measured by liquid chromatography-tandem mass spectrometry (LC-MS/MS). (**a**) Progesterone, (**b**) 17-OHP, (**c**) androstenedione, (**d**) testosterone, and (**e**) estradiol from the culture with the tissue of the 7-year-old patient. (**f**) Progesterone, (**g**) 17-OHP, (**h**) androstenedione, (**i**) testosterone, and (**j**) estradiol from the culture with the tissue of the 12-year-old XIAP deficiency patient. Blue, culture condition A; orange, condition B; grey, condition C. Hormone levels were normalized according to the number of tissue pieces in the culture dish at the corresponding time points. For an intuitive understanding, the diagrams are presented on the same size scale for the 7- and 12-year-old patient.

To study cellular functionality and secretory competence of the LCs in the used organ culture medium, testosterone was initially analyzed for the 7-year-old boy and the 9-year-old SCD patient, applying an ELISA assay. Here, measurable values were only obtained for the 7-year-old patient, whereas absorbance remained at zero when testosterone in the culture medium of the 9-year-old SCD patient was investigated. Due to a difficulty with the ELISA method, it turned out that the standards included in the assay did not produce a calibration curve suitable for the value range of the prepubertal samples, so accurate calculation could not be reached utilizing this approach. As an alternative strategy, LC-MS/MS was applied with the additional advantage of simultaneous determination of testosterone and a variety of other steroid hormones. In Figure 9, the levels of testosterone, progesterone, 17-OHP, androstenedione, and estradiol are depicted, and due to the presumably age-related differences in the secretion level, these are shown individually for each patient. 

In the adult testis, testosterone and precursor hormones are synthesized by the LCs. In this process, progesterone, which itself constitutes the precursor for 17-OHP, is produced from pregnenolone. In turn, the 17-OHP represents an intermediate in the synthesis of several hormones such as cortisol, estrogen, and testosterone. Within the testosterone production pathway, it is first converted into androstenedione, which is then either transformed into testosterone or metabolized by the CYP19A1 aromatase into estrone, the precursor for estradiol. Testosterone may also be converted to estradiol by CYP19A1 aromatase [51,52].

Organ culture experiments on the testis tissue of the 7-year-old patient and the 12-year-old XIAP deficiency patient revealed that under the selected culture conditions, LCs are capable of producing testosterone and its precursor hormones over the entire cultivation period (Figure 9), with the exception of androstenedione under culture conditions A and B in the case of the first patient (Figure 9c). Overall, it can be seen that higher hormone concentrations are achieved in the organ culture of the 12-year-old patient (Figure 9f–j vs. Figure 9a–e), presumably for age-related reasons. An increase in hormone levels from day 1 to 10 is seen in both patients. However, certain fluctuations are visible over the entire period, probably on the one hand due to the sample extraction every 7 days and on the other hand due to the feeding interlude, where half of the medium was changed every 2 days.

When comparing the three different culture conditions, patient-specific differences come to light. In the case of the 7-year-old patient, highest hormone values were detected in condition C, while the lowest were detected in condition A (grey bars vs. blue bars in Figure 9a–e). Considering the 12-year-old patient, it is basically the opposite (Figure 9f–j): Condition A turned out to be the best regarding the androstenedione and testosterone levels (Figure 9h,i), as well as the progesterone concentration starting from day 11 (Figure 9f) and the values for 17-OHP beginning from day 9 (Figure 9g). In contrast, estradiol from the culture of the 12-year-old was highest under condition C (grey bars in Figure 9j) and mostly lowest (with one exception on day 19) under condition A (blue bars), similar to the 7-year-old patient (Figure 9e). In terms of androstenedione in the case of the 7-year-old patient, measurable levels could only be obtained for condition C on days 5, 7, 11, and 15 (Figure 9c).

Even though only a few patients were examined in this study and the values therefore must be considered with caution, in comparison between conditions B and C, higher testosterone production is achieved under condition C. This applies to all time points for the tissue of the 7-year-old patient (Figure 9d) and to the time points up to culture day 17 for the tissue of the 12-year-old patient (Figure 9i).

To determine linear and non-linear dependencies between testosterone production and the secretion of its precursors, progesterone, 17-OHP, and androstenedione, and the metabolite estradiol in the organ cultures, linear regression and Spearman correlation analyses were performed. Here, testosterone levels over the cultivation time were compared for each of the two patients in turn with the concentrations of the other steroid hormones. Only those measurement series were included where none of the values were below the detection level. In the case of the 7-year-old patient, therefore, androstenedione for all conditions and estradiol for conditions A and B were ruled out. Accordingly, for the 12-year-old patient, the androstenedione concentrations for condition B were omitted.

**Figure 10:**
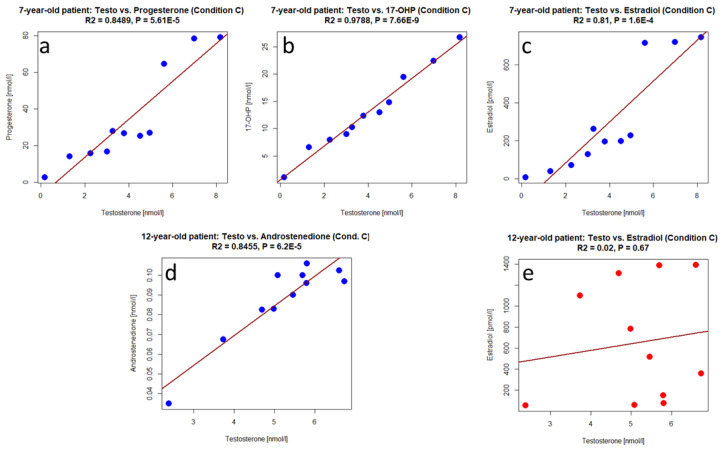
Linear regression for the steroid hormone levels. (**a**) Comparison of testosterone vs. progesterone, (**b**) testosterone vs. 17-OHP, and (**c**) testosterone vs. estradiol under culture condition C from the experiments with the tissue of the 7-year-old boy. (**d**) Comparison of testosterone vs. androstenedione, and (**e**) testosterone vs. estradiol under condition C from the experiments with the tissue of the 12-year-old boy. R^2^ values leading to significant relations are shown in blue, and those leading to non-significant associations are shown in red.

Linear regression and Spearman correlation analyses indicated strong dependencies between the variables in the 7-year-old patient in almost all cases. Figure 10a–c displays some examples for linear regression analysis. These results, on the one hand, demonstrate a direct dependence of testosterone on its precursor hormones and, on the other hand, indicate that, at least under condition C, probably an equal proportion of the testosterone produced is converted into estradiol.

Regarding the organ culture of the 12-year-old patient, testing indicated strong to moderate dependencies in 8 of 11 cases (see Figure 10d for an exemplary representation). In this context, it should be mentioned that in this experiment, technical problems with the incubator occurred after the culture period of approximately 2 weeks, leading to a gradual CO_2_ increase until the problem could be fixed on culture day 19. In order to exclude the influence of these interferences on the association of hormone levels, tests showing only weak dependencies from the series of measurements over 21 days were repeated with the values up to the time point of 15 days. This actually led to a significant dependency in 2 out of 3 previously non-significant cases. An exception was the comparison between testosterone and estradiol under condition C, where no significant association could be found (Figure 10e). The latter finding could be interpreted to mean that proportionally more testosterone was converted to estrogen in condition C than in conditions A and B or in the organ culture from the 7-year-old patient.

### 3.9. Morphological Evidence of the LCs by Immunofluorescence Staining, Semithin Technique and TEM

Immunostaining was performed for the LC markers STAR, CYP17A1, and INSL3. STAR and CYP17A1 are both key enzymes within the male hormone steroid synthesis cascade. While STAR acts as an enhancer in the conversion of cholesterol to pregnenolone, CYP17A1 functions in the transformation of pregnenolone to progesterone. INSL3 represents a marker for adult, and thus mature LCs. In contrast to INSL3, STAR is also already expressed by more immature or dedifferentiated LCs [53].

Regarding the native tissues of our samples, STAR-positive LCs could be encountered in the case of both patients. In terms of CYP17A1, the outcome was similar, although in the case of the 7-year-old patient, not all areas of the tissue presented CYP17A1-positive cells. In contrast, INSL3 was absent in the native tissue of the 7-year-old boy, whereas the tissue of the 12-year-old patient was found to be highly heterogeneous with respect to the quantity of the INSL3 signals (Figure S6d vs. Figure S6a–c). Here, areas with very few INSL3 signals could be detected next to areas with more frequent INSL3 signals (Figure S6a vs. Figure S6b,c). 

The cultured samples of the 12-year-old patient displayed some INSL3 signals in all conditions until the end of the culture time (Figure S6e–k), while only a few weak INSL3 signals were present in the tissue of the 7-year-old boy. This finding is consistent with the lower level of steroid hormones detected in the culture medium in the case of the younger patient. Adult testicular tissue was used as a positive control (Figure S6h), whereas cultured prepubertal testicular tissue served as the negative control (Figure S6l).

The development of STAR expression during the culture was difficult to follow since over the time, signals increasingly also appeared in tubular cells, a fact that we could not explain (Figure S10a). A similar staining phenomenon with tubular cells positive for the LC marker occurred using another INSL3 antibody (Figure S10b), in which the positive control from the adult testis and the negative controls each, likewise, showed a correct staining result.

CYP17A1 immunofluorescence staining over the cultivation period indicated increasing expression in the tissues of both patients. Consistent with the higher hormone levels in the organ culture of the 12-year-old patient, stronger CYP17A1 signals were detected in the tissue of this patient compared to the tissue of the 7-year-old patient (Figure S7a vs. Figure S7b). In the case of the 7-year-old patient, the most intensive CYP17A1 signals were found in tissue cultured under condition C, also fitting the steroid hormone measurement values.

Especially remarkable in the case of the 12-year-old patient is that one of two tissue pieces cultured for 3 weeks under condition A particularly showed stronger CYP17A1 signals compared to all (other) conditions and time points. This might explain the high progesterone peak under condition A at day 15 (Figure 9d) and the increase of subsequent steroid hormone product levels (Figure 9b,f,h). Also, in regard to the CYP17A1 staining, however, partial protein expression in some tubular cells was also observed (Figure S10c).

**Figure 11:**
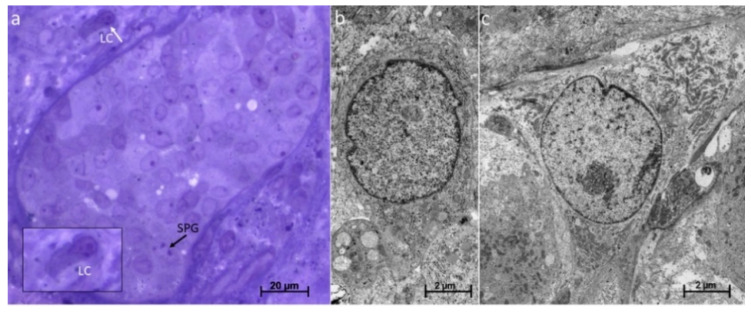
Leydig cells (LCs) after 3 weeks of organ culture. Prepubertal testis tissue from the 12-year-old XIAP deficiency patient. (**a**) Toluidine blue pyronin-stained semithin section from the tissue cultured under condition C. LC marked with white arrow. Doublet of spermatogonia (SPG) marked with black arrow. (**b**) TEM micrographs of LCs in the tissue cultured under condition B, and (**c**) under condition C show the LC typical polygonal cell body structure containing cytoplasm rich in endoplasmic reticulum and a round nucleus whose nuclear membrane is lined by a thin layer of condensed chromatin as described in [54]. Scale bars: 20 µm (**a**), and 2 µm (**b**,**c**).

For further identification of the LCs in the cultured tissues, two tissue sections from the 12-year-old patient, cultured for 3 weeks (conditions B and C), were examined using semithin sections. As a result, LCs could be detected in both pieces of the tissue. Figure 11a, exemplarily, shows a LC in a semithin section from condition C. From the Epon-embedded tissue pieces, ultrathin sections were subsequently prepared for the evidence of the LCs at the ultrastructural level. By electron microscopic visualization via TEM, LCs could be found in the cultured tissue from both conditions (Figure 11b,c). However, due to the limited number of samples, only these two conditions from the 12-year-old patient could be subjected to this evaluation.

### 3.10. Prepubertal SPG from Boys 7 Years of Age and Older and Adult SPG Exhibit Similar Mitochondrial Sizes

Based on the transferability of SPG- and organ-culture-conditions from adult to prepubertal tissue, regarding patients of a certain age group demonstrated by our experiments, and therefore to compare key morphological characteristics important for the metabolism of SPG on the ultrastructural level, we used TEM to examine the mitochondrial size of prepubertal SPG compared to adult A-SPG. For this purpose, eight prepubertal testicular tissues from boys of different ages (between 1.5 and 13 years) and two tissues from adult males, showing complete spermatogenesis (see Table 2), were selected. Undifferentiated SPG were chosen on the basis of the typical morphology of undifferentiated SPG (e.g., A dark and A pale SPG) and the appearance of electron-dense intermitochondrial cement, not present in B-SPG [55,56,57,58,59,60].

**Figure 12:**
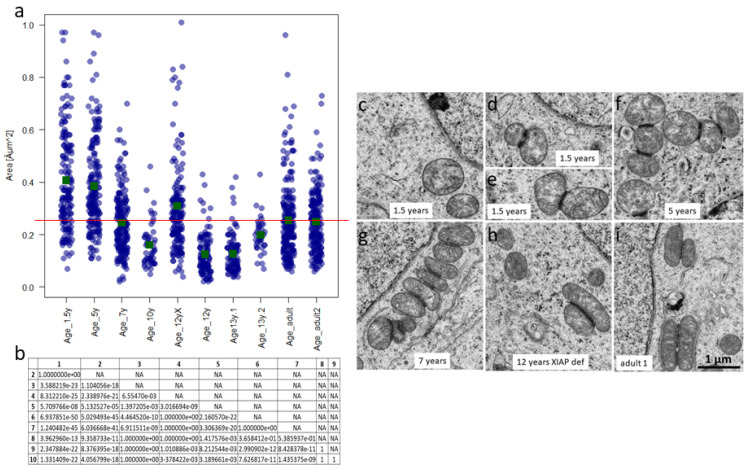
Mitochondria (MT) in undifferentiated spermatogonia (SPG) from prepubertal and adult patients. (**a**) Mitochondrial size measured as surface area [µm^2^] from TEM micrographs. Each blue ball represents the size of one mitochondrion. The mean is indicated as a green square, with the red line reflecting the means from the adult testis tissue. (**b**) *p*-values from pairwise *t*-test with Bonferroni multiple-comparison correction. NA, not applicable. (**c**–**f**) TEM micrographs of MT from early prepubertal SPG, and (**g**,**h**) late undifferentiated prepubertal SPG vs. (**i**) adult undifferentiated SPG. Scale bar: 1 µm.

In total, the sizes of 1023 MT of prepubertal SPG and 428 MT of adult SPG were determined. Our measurements and statistic calculations via pairwise *t*-test plus Bonferroni correction revealed that on average, the MT in the SPG of the two younger patients (1.5 and 5 years old) were of significantly larger size (mean 0.41 and 0.38 µm^2^, respectively; Figure 12a, 1st and 2nd patient on the left, and Figure 12b) compared to the MT of the older prepubertal boys (7, 10, 12, and 13 years old; 3rd to 8th patient in Figure 12a) and those from the adult men; Figure 12a, last two patients). In these two latter cases (older prepubertal patients and adult patients), no higher surface area values than 0.25 µm^2^ on average were measured (see the red line as a kind of threshold in Figure 12a). An exception to this was the one 12-year-old patient exhibiting XIAP deficiency, a disease associated with apoptosis dysfunction (Figure 12a, 5th patient). In the case of this patient, it should be noted that, in addition to similar mitochondrial sizes compared with the older prepubertal patients from the age of 7 years, the jitter plot also indicates values in the upper third of the graph, as found in the SPG of the two younger prepubertal patients.

Figure 12c–i illustrates the crucial criteria of the MT in the SPG of the patients from the corresponding age groups: The younger patients not only display larger, but also considerably rounder MT (Figure 12c–f), whereas the patients with the smaller MT often show these in a more elongated shape (Figure 12g–i).

The presence of the larger MT in the case of the XIAP-deficient patient (shown in the upper third of the graph in Figure 12a) could certainly be explained in the context of the underlying disease; due to the apoptosis impairment, MT from earlier prepubertal stages can persist to a later prepubertal stage. Indeed, the SPG with the larger MT in this case also exhibit shapes as round as those of the two younger children (Figure S11).

## 4. Discussion

In the 1st part of the study, three prepubertal tissues from boys of different ages (7, 9, and 12 years) as well as different diseases were cultured under three different conditions based on the non-xeno-free StemFlex culture medium, which we had found to be the most favorable culture medium for adult human organ culture in comparison to Nutristem^®^hPSC XF (Biological Industries, Kibbutz Beit Haemek, Israel) and StemPro^®^-34 SFM (Life Technologies Corporation, Grand Island, NY, USA) (Figure S12). StemFlex medium plus the related StemFlex supplement with Pen/Strept served as the basic condition (condition A) for our experiments, whereas conditions B and C were further complemented with FSH and LH as well as mainly stemness-promoting factors. The aim of this part of the study was to test whether, with the culture conditions used, SSCs could be maintained as a basis for developing a model for human in vitro-spermatogenesis and further investigate the effect on the other testicular cell types under the selected organ culture conditions.

Activin A and nodal represent pleiotropic growth factors of the TGFß superfamily. By binding to the same receptors, their ligands signal via the same pathway, resulting in the activation of the SMAD transcription factor family, which in turn is implicated in downstream actions such as cellular stemness, proliferation, and cell cycle regulation [61,62,63]. In a single cell study [64], investigating the dynamic transcriptional profile of the developing human testis, the activin A signaling pathway was found to be a candidate key player during human puberty, since activin A receptors are expressed in SPG and their key inhibitors specifically in undifferentiated type 0 and type 1 SPG. Sato et al. (2011) [28], used activin A as a stimulatory molecule for induction of murine in vitro-spermatogenesis in a neonatal organ culture model. Activin A is ascribed numerous other functions in the testis, including SC differentiation and the determination of steroid hormone levels [65,66,67,68]. Nodal, which in cell/organ culture is often used in conjunction with activin A [69], is expressed in the cytoplasm of SPG in the adult human testis, including A dark-SPG (data from Human Protein Atlas) [70,71]. In the fetal testis, activation of the nodal signaling pathway impacts androgen production, including the testosterone steroid precursor hormones androstenedione and 17-OHP [69]. FGF2 and GDNF constitute known growth factors commonly used in cell culture to promote the self-renewal capacity of SPG and SSCs. FGF2 thereby only acts in interaction with GDNF, enhancing its efficacy [72,73]. GDNF, furthermore, was found to be significant for the maintenance of prepubertal human SPG in cell culture [74]. CSF1 (M-CSF), likewise a growth factor, was utilized for induction of murine adult in vitro-spermatogenesis by Sato et al. in 2015 [75]. In the testis, it is expressed by the interstitial macrophages, the PTCs and the LCs, and is considered an extrinsic stimulator of mouse SSC self-renewal by shifting the ratio of SSC renewal vs. differentiation in favor of spermatogonial self-renewal [72,76,77]. Its receptor, CSF1R, inter alia, is expressed on the surface of SPG where it promotes their proliferation and self-renewal capacity [76,78,79]. Since CSF1R was also found to be expressed by human LCs, a link between CSF1 and the testicular paracrine/autocrine system was postulated [79].

The effects of the organ culture conditions on the individual cell populations over time are summarized in Table 3 and discussed in the two following text sections.

### 4.1. Effect of the Organ Culture Conditions on the Germ Cell Populations

Within our organ culture approach, DDX4-positive germ cells and PIWIL4-positive SPG could be maintained throughout the entire cultivation period in the tissue of the 7-year-old and the 12-year-old patient (Figure 4 and Figure 5). Nevertheless, a decreasing trend in the counts of both cell types was observed. The decline in the number of the SPG during testicular prepubertal organ culture is a common and recurrent observation in the studies published in this field so far [20,21,22,23,24,25], summarized in Supplementary Table S3. The results from all these studies are difficult to compare, since for the detection of the SPG, different markers were used and different related units for the cell counting approaches were applied (e.g., cells/well preserved tubules, cells/round tubules, cells/mm^2^). In our case, we quantified the SPG in the tubular areas of the tissue pieces and measured in “cell count/mm^2^”. Therewith, the cell number is related to the whole tissue piece and only fibrous areas are excluded. In this way, the quality of the entire cultured sample is considered, which is not taken into account when, e.g., only the “well-preserved tubules” are determined [20,21]. As a marker, de Michele et al. used MAGEA4 to identify SPG. In our case, the pan-germ cell marker DDX4, and the SPG marker PIWIL4 were utilized [40,80,81]. Considering the fact, that the most advanced germ cells in this tissue were (with very few exceptions) SPG, we value our DDX4 staining as comparable to the staining for the marker MAGEA4. Medrano and colleagues analyzed the number of UTF1+, c-KIT+ (KIT), and VASA+ (DDX4) germ cells per tubule cross section [22]. Interestingly, here an increase in UTF1+ cells per tubule cross-section in one condition was reported, while DDX4+ cell counts remained half as high as at the start of the experiment after two weeks [22] (extracted from the graph, Figure 3c,h). The UTF1+ population usually should also express DDX4. In our study, we additionally noticed UTF1 expression in SCs, albeit to a lesser extent. Here, the question arises whether UTF1-positive SC cell nuclei may have been co-quantified as SPG in [22]. Portela and colleagues used the percentage of tubules with MAGEA4+ SPG for quantification [23]. Kurek et al. measured the germ cell count as DDX4+ cells per round tubule [24]. Wang and colleagues used an MAGEA antibody as well as GAGE and DDX4 as markers for the SPG [25]. The samples here were only analyzed on days 0 and 60.

In our study, double staining of the germ cell markers with the proliferation marker Ki-67 revealed preservation of germ cell proliferation over the whole culture time, whereby the numbers of Ki-67+ germ cells also decreased (Figure 5a). De Michele et al. used MAGEA4 and Ki-67 to identify proliferating SPG [20,21]. In both studies, a reduction in the quantity of proliferating SPG was noticed. Portela et al. used MAGEA4 and the proliferating cell nuclear antigen (PCNA) to determine SPG proliferation [23], with the latter representing a protein that is also involved in DNA replication and DNA repair, which is why its expression does not exclusively label proliferating cells. The result of this study was that the SPG remained proliferative until the end of the experiment, but the percentage of tubules containing at least one proliferating spermatogonium strongly decreased within the first three weeks. The studies by Kurek et al. and Wang et al. also used the proliferation marker Ki-67 [24,25], but not in a costaining with an SPG-specific marker. 

The most advanced germ cells after culture in our study were SPG. In single tissue sections, a few BOULE+ cells were present that we believe to be remnants from before the culture. De Michele et al. detected BOULE+ SPCs in all sections and, in one single case, elongated spermatids expressing the angiotensin-converting-enzyme (ACE) [21]. Medrano and colleagues found an increase in the premeiotic DDX4+/SYCP3+ germ cells until day 14 [22]. However, further advanced stages of spermatogenic cells were not observed. Portela and colleagues reported no advanced germ cells after their cultivation approach [23]. Wang et al. found BOULE+ SPCs after 60 days in one condition, supplementing retinoic acid [25]. A possible factor contributing to the stronger differentiation potential of the germ cells may be the age of the donors. The cultivated tissues used by Wang and colleagues were more juvenile in comparison to the other studies [25].

Comparing our three organ culture conditions in terms of number of the germ cells and SPG maintained over the cultivation period, condition C performed best in 9 out of 12 cases. However, statistical significance could not be demonstrated, which most likely results from the low number of samples used in this study. Due to the small sample size, these results should be regarded with caution.

### 4.2. Effect of the Organ Culture Conditions on the Somatic Cell Populations

Testicular somatic cells include SCs, LCs, and PTCs. While the SCs constitute a fundamental component of the SSC niche, the interstitial LCs are responsible for male steroid hormone secretion. The PTCs, on the other hand, enclose the outside of the seminiferous tubule basement membrane and represent the production site for the extracellular matrix proteins [73]. During our organ culture, maintenance and/or maturation of all testicular cell types examined were observed.

For the PTCs, the markers LAMA1 and alpha-SMA were employed. According to the study by Kurek et al. [24], the presence of the glycoprotein LAMA1, as an extracellular component of the basement membrane of testicular seminiferous tubules, is a basic requirement for the maintenance of SPG and SSCs in human prepubertal organ culture. In mammals, LAMA1 expression starts in early embryonic development, where it appears in epithelial morphogenesis. In some tissues, LAMA1 expression is retained in the adult stage, where it is expressed by the epithelial and endothelial cells [82,83]. In humans, expression of this protein is established six weeks post-conception [24]. In the adult human testis, LAMA1 presents a part of the secretome of the testicular PTCs [84]. In our experiments, the expression of LAMA1 was maintained under conditions B and C until the end of the culture. In contrast, in condition A, LAMA1 expression was reduced to approximately 50% (Figure 3c,l,m). This fits the low value of the preserved DDX4-positive cells under condition A after 3 weeks (1.7 cells/mm^2^; Figure 5a) and also the data of the Kurek study [24].

For the assessment of the maturation status of the prepubertal tissue and as a standard marker for the testicular PTCs, alpha-SMA was utilized. Alpha-SMA represents an excellent marker for assessing the prepubertal status of testicular tissues in the non-human primate species marmoset, and partial expression of this protein marks an ongoing process of PTC differentiation, which is still incomplete [38,39]. Before culture, alpha-SMA protein was mainly seen in the vascular regions of the tissue and hardly detectable within the seminiferous tubule basement membrane. After one cultivation week, we could observe SMA protein expression predominantly within the basement membranes, signaling ongoing PTC maturation. These data are consistent with the findings of Medrano et al., who detected a progressive increase of the ACTA2 transcript during testis organ culture using PCR, with ACTA2 representing the gene for alpha-SMA protein [22]. Our findings fit the results of Wang et al. [25], who saw alpha-SMA protein expression in the PTCs in three of four cultured tissues from younger boys (0.5–1.4 years) at organ culture day 60. In our approach, a difference between the three culture conditions could not be determined, suggesting that the added hormones and growth factors in the organ culture did not contribute essentially to the in vitro-PTC maturation.

The markers SOX9 and CLDN11 were applied to assess the maturation status of the SCs. While SOX9 is expressed in mature SCs and also in the adult human testis, CLDN11 represents a component of the BTB, which is necessary for SC tight junction formation [85]. CLDN11, likewise, is expressed in the human adult state. As we detected consistent expression of SOX9 and CLDN11 before, during, and after the organ culture, signaling a certain maturation state, no markers for immature SCs were utilized. Since weak UTF1 expression in the SC nuclei, however, might indicate some cellular immaturity, we performed an ultrastructural examination comparing SC characteristics before and after the culture. Here, actual differences came to light: SCs from the cultured tissues tended to have more lobulated nuclei and more prominent nucleoli, suggesting a more mature status induced by the organ culture conditions. Nevertheless, because only the tissues from the 12-year-old patient and only those from conditions B and C could be included in the investigation, the data situation for general statements is too weak.

LC maturation over the culture period was determined using various cell type-specific markers. STAR could be detected in the native tissues of both patients, whereas CYP17A1 was also present in the tissues of both patients, but to a much weaker extent in the case of the 7-year-old patient. INSL3 was only seen in the tissue of the 12-year-old patient, and here there is great heterogeneity in terms of the number of the INSL3 signals. During the organ culture, tissues from both patients showed an increase in CYP17A1 protein expression, although this was much more pronounced in the case of the 12-year-old patient. In the tissue of the 7-year-old patient, the strongest CYP17A1 signals could be detected in condition C. The situation was similar with regard to the marker INSL3: The cultured tissue from the 12-year-old boy displayed INSL3 signals in all areas, whereas in the tissue of the 7-year-old boy, INSL3 protein expression was seen only at a few sites. The presence of the LCs in the tissue cultured for three weeks was simultaneously demonstrated by means of semithin sections and at the ultrastructural level (Figure 11). However, only tissue from the 12-year-old boy and only tissue from conditions B and C could be used here.

In addition to the examination of the LCs at the morphological and protein expression level, steroid hormone production was quantified in the organ culture supernatant by LC-MS/MS as a functional proof. Here, the concentration of testosterone and its precursor hormones, progesterone, 17-OHP, and androstenedione, as well as estradiol, was measured. Overall, the steroid synthesis and conversion validated the endocrine function of the tissues in our organ culture approach. In both of the patient samples that were used for the organ culture experiments, a dynamic in hormonal secretion is clearly visible. The detailed breakdown of steroids enables us to retrace steroid enzyme function in the testis. In Figure 9, only the measurable levels are displayed. Additionally, the adrenal-derived steroid hormones aldosterone, DHEAS, cortisone, and cortisol were analyzed. As we had expected, no aldosterone was synthesized, as the required enzyme, cytochrome P450 family 11 subfamily B member 2 (CYP11B2), is only expressed in the adrenal cortex. Interestingly, low levels of cortisol, which enzyme is also absent in the testes, were detected in some samples. The substance did not show a trend and was also found in the media before cultivation. We observed an increased secretion of testosterone in both organ cultures, regardless of LH substitution. However, absolute levels of testosterone and especially the progenitor steroids in the 12-year-old were higher than those in the 7-year-old, as the maturation of the tissue was probably more advanced. Even though the testicular biopsy did not include SPCs or further developed germ cells, it produced up to six times more testosterone at peak (21.5 nmol/L, day 21). Interestingly, one sample from the 12-year-old patient subjected to the condition with no added factors peaked highest in testosterone on day 21 and contained the lowest levels of estradiol. Unequal distribution of INSL3-expressing LCs in the tissue of this patient could be an explanation for the high testosterone (see Figure S6a vs. Figure S6b,c). However, higher estradiol levels would be more plausible. Such cases need to be verified with larger sample numbers. Testosterone secretion of the human prepubertal organ cultures was also reported in the other studies where this was measured (Supplementary Table S3). For this purpose, ELISA assays were used.

Estradiol, which is also included in our mass spectrometry, is synthesized by converting testosterone via aromatase. In almost all cases, high estradiol levels were observed with high testosterone. Sharma et al. (2022) reported similar results comparing high-dose and low-dose exposition to human chorionic gonadotropin (hCG) and FSH in a marmoset microfluidic setup [39]. In our experiments with the tissue of both patients, estradiol secretion was dependent on the culture condition too, with the highest values shown in condition C, supplemented with FSH, LH, and CSF1, in addition to the other growth factors. The differences regarding the culture condition with the highest steroid hormone production between the patients might be due to the different maturation status of the LCs. 

The higher testosterone secretion in condition C compared to condition B, even though only tissue from a few patients could be examined, fits with the data of the Pollard study which showed that mice testicular macrophage depletion, and thus a CSF1 absence, leads to a reduction in the testosterone levels [86].

In our study, the stimulability and thus functionality of the LCs was demonstrated by the determination of the steroid hormone profile by LC-MS/MS. This is the first study that beside testosterone secretion, also considers the production of the testosterone precursor hormones in human prepubertal testicular organ culture.

### 4.3. Heterogeneity of the Prepubertal Testis Tissues with Regard to the Proportion of DDX4+ Germ Cells and PIWIL4+ SPG

The prepubertal testicular tissues used (1st part of the study) and those which were further examined (2nd part of the study), were found to be highly heterogeneous with respect to the germ cell (DDX4+) and spermatogonial cell (PIWIL4+) quantities: The total DDX4+ cell counts varied between approximately 70 and 1050 cells/mm^2^ (Figure 6g), whereby the patient with SCD displayed the lowest DDX4+ germ cell count. SCD is known to reduce the spermatogonial cell number, even without gonadotoxic hydroxyurea treatment [87,88]. Our patient was subjected to hydroxyurea for seven years. In SCD patients treated with hydroxyurea, germ cell numbers are significantly reduced [89]. Contributing to that, Portela and colleagues (2020) described an impaired spermatogonial DNA methylation pattern in patients exhibiting SCD [23]. In our case, the organ culture of the patient with SCD was discontinued after two weeks due to the almost complete depletion of germ cells. Consequently, it seems unlikely that in vitro-spermatogenesis can be achieved with the tissue of such a patient. Hence, autologous grafting of undissociated testicular tissue after gonadotoxic therapy might be the only option for fertility restoration in patients with sickle cell anemia receiving hydroxyurea.

Calculating the differences between the amounts of DDX4+ cells (Figure 6g) and PIWIL4+ cells (Figure 6h) at the individual patient level, a gap can be seen (Figure 6i): The patient with SCD and the patient with MDS are displaying almost the same amount of DDX4+ cells as PIWIL4+ SPG, hinting at a germ cell composition of mainly undifferentiated SPG. Both the 7-year-old patients with thalassemia and the 12-year-old XIAP deficiency patient show the same difference between the DDX4+ and the PIWIL4+ cells, so it can be assumed that the SPG differentiation, including proliferation of the SPG, is already more advanced here. The same difference between DDX4+ and PIWIL4+ cells of approximately 100, seems to indicate a similar state of the spermatogonial differentiation. On the other hand, the 12-year-old patient with hyper-IgE syndrome presents 700+ more DDX4+ than PIWIL4+ cells. Since hardly any SPCs were found in the semithin section in the case of this patient (see Table 1 in Materials and Methods), the result found in the latter case possibly indicates a less disease-related, but more age-appropriate stage of spermatogenesis with more differentiating and proliferating SPG (66 DDX4+/Ki-67+ cells/mm^2^, see the penultimate pink bar in Figure 6g). It is difficult to determine whether age- or rather disease-specific differences among the patients are reflected here, as well as in the cases of the other patients, or whether coincidental similarities are reflected. If correlations actually exist, they can only be clarified by large-scale studies involving a number of similar diseased patients of different age groups.

### 4.4. Comparison of the Mitochondrial Sizes

Since the 1st part of our study showed the feasibility of organ culture conditions developed on human adult testicular tissue, for prepubertal testis tissue from patients 7 years of age and older, with respect to SPG/SSCs preservation, we aimed on the identification of similarities in the ultrastructure of the MT from adult and prepubertal undifferentiated SPG. MT represent the key components in the metabolism of a cell, and a recent study [90] demonstrated that during porcine male prepubertal development (between one and eight months of life), the SSCs undergo a metabolic transition from mitochondrial respiration to anaerobic glycolysis, different from mice, where this metabolic switch occurs prenatally. As glycolysis takes place in the cytoplasm of cells, in contrast to the tricarboxylic acid cycle and oxidative phosphorylation occurring within the MT [91], it is to be expected that cells whose energy production depends on oxidative phosphorylation display MT of a larger size than cells whose metabolism takes place in the cytoplasm. For this reason, prepubertal SSCs before metabolic transition should be equipped with a larger MT than prepubertal SSCs after metabolic transition. To examine this fact in more detail using the human model, we measured the mitochondrial sizes of undifferentiated SPG from younger and older prepubertal boys and compared these with the mitochondrial sizes of undifferentiated SPG from adult males. Our investigations revealed high statistically significant commonalities in the mitochondrial sizes between adults and prepubertal boys from an age of 7 years (see Figure 12b, patients 1 and 2 vs. patients 3–10, and Figure 12a). These data might hint at the approximate timing of the metabolic transition from mitochondrial respiration to anaerobic glycolysis in human SSCs, which is unknown so far. According to the data from the Voigt study [90], the SPG with the larger, rounder MT of the younger children might be interpreted as early prepubertal SSCs, and those with the smaller, more elongated MT of the older children as late prepubertal SSCs. However, patient-specific differences and disease-related abnormalities in spermatogenesis in the patients involved in this study should not be disregarded, and the 5-year-old boy with MSD may not be representative for his age group. The presence of even smaller mitochondrial sizes in the SPG from boys aged 10 to 13 years (see Figure 12a,b, patients 4, 6, 7, and 8) compared with the 7-year-old boy (patient 3) and the two adult patients (patients 9 and 10) could present a “levelling off of the MT sizes toward an adult mitochondrial size level”.

These findings indicate that different culture conditions regarding oxygen concentrations might be useful for the in vitro-cultivation of prepubertal human SSCs from patients of different ages. Further research on this topic, including large-scale studies closely covering the entire male prepubertal age range and single cell analyses, could be helpful to complement the data presented here.

## 5. Conclusions

The culture conditions used in this study promote SPG maintenance and proliferation as well as the maturation of the prepubertal testicular somatic cells. The LCs, furthermore, could be stimulated for testosterone and precursor hormone production. FSH, LH, and growth factors, especially CSF1, exerted positive effects on the cultured cells, although due to the small number of samples applied, no significant effect could be reported. Due to the small sample size, the results from the cultivation experiments should be viewed with caution. Further optimization in regard to the cultivation of SPG and SSCs in boys aged seven years and older might be achieved by application of the culture conditions under hypoxia, as indicated by the ultrastructure of MT in the undifferentiated SPG of patients from this age group.

## Figures and Tables

**Table 1:** Patients whose tissues were applied for organ culture and further germ cell quantification.

Patient Age	Diagnosis	Serum Testosterone	Most Advanced Germ Cells Type			Tissue Used for
[Years]		[µg/L]	Epon Embedded Tissue		Paraffin Embedded Tissue	(a) Organ Culture,
			Semithin Section	TEM	Immunostaining	(b) Quantification
7	ß-Thalassemia major	<0.07	SPG	SPG	SPG, rare cases of SPCs	a, b
12	XIAP deficiency	<0.07	SPG	SPG	SPG	a, b
9	Sickle cell disease	<0.07	rare cases of SPG	n.d.	rare cases of SPG	a, b
7	ß-Thalassemia major	0.08	SPG	n.d.	SPG	b
9	Myelodysplastic syndrome	0.08	SPG	n.d.	SPG	b
12	Hyper IgE syndrome	0.13	SPG, rare cases of SPCs	SPG, rare cases of SPCs	SPG	b

A depiction of the histology of the tissues used for organ culture in the native state is shown in Figure S1. SPG, spermatogonia; SPCs, spermatocytes; n.d., not determined.

**Table 2:** Patients for mitochondrial size measurements.

Patient Age	Diagnosis	Serum Testosterone	Most Advanced Germ Cells Type
[Years]		[µg/L]	Semithin Section
1.5	CD40L deficiency, Hyper IgM syndrome	<0.07	SPG
5	Myelodysplastic syndrome	<0.07	SPG
7 ^a, b^	ß-Thalassemia major	<0.07	SPG
10	ß-Thalassemia major	<0.07	SPG
12 ^a, b^	XIAP deficiency	<0.07	SPG
12 ^b^	Hyper IgE syndrome	0.13	SPG, rare cases of SPCs
13	Glioblastoma	0.5	SPG, SPCs
13	Leukocyte adhesion defect	<0.07	SPG, SPCs
adult, 39	Status post vasectomy	n.d.	SPTs
adult, 47	Obstructive azoospermia	5.77	SPTs

Indices in the first column: Tissue was also used for “a” organ culture and “b” for germ cell quantification. SPG, spermatogonia; SPCs, spermatocytes; SPTs, spermatids; n.d., not determined.

**Table 3:** Main results of the organ culture.

Cell Type	Method/Marker	Interpretation	Differences between Conditions
**SPG/SSCs,**	IFL/PIWIL4,	Maintenance, cell count reduced	slightly better in C after 3 wks
**germ cells (GCs),**	DDX4,		
**proliferating GCs**	DDX4 + Ki-67		
**SPCs**	IFL/BOULE	7-year-old patient:	7-year-old patient:
		rare cases of cells,	native tissue: 1-2 cells
		cells present before culture	1 week: few cells in A + C
		12-year-old patient:	12-year-old patient:
		rare cases of cells,	native tissue: no cells
		cells most likely present	1 week: few cells in A + B
		before culture	2 weeks: few cells in B
**PTCs**	IFL/SMA,	Cellular maturation	no
	LAMA1	Preservation of expression	50% reduced in A after 3 wks
**SCs**	IFL/SOX9	Maintenance	no
	CLDN11	BTB maintenance	no
	TEM/ultrastructure	Maturation	only B + C tested
**LCs**	LC-MS/MS/	Functionality of cells proven with	7-year-old patient:
	progesterone,	steroid hormone production	best condition: C
	17-OHP,	in the tissues of the 7-year-old	
	androstenedione,	and the 12-year-old patient,	12-year-old patient:
	testosterone,	higher levels in the	best condition: A
	estradiol	case of the 12-year-old patient	
	ELISA/testosterone	9-year-old SCD-patient,	no secretion
		no testosterone	
	IFL/STAR,	Expression present	no
	CYP17A1,	Increasing expression (7-year-old boy: lesser extent)	7-year-old patient, best condition: C
	INSL3	Expression present in the tissue of 12-year-old boy	no
	Semithin/morphology	Presence of LCs at the morphological level confirmed	no; condition A n.d.
	TEM/ultrastructure	Presence of LCs at the ultrastructural level confirmed	no; condition A n.d.

SPG, spermatogonia; SSCs, spermatogonial stem cells; GC, germ cells; SPCs, spermatocytes; PTCs, peritubular cells; SCs, Sertoli cells; LCs, Leydig cells; IFL, immunofluorescence staining; TEM, transmission electron microscopy; LC-MS/MS, liquid chromatography-tandem mass spectrometry; 17-OHP, 17-hydroxyprogesterone; ELISA, enzyme-linked immunosorbent assay; SCD, sickle cell disease; wks, weeks; n.d., not determined.

## Data Availability

Data from this study are available from the corresponding author upon request.

## Supplementary Materials

The following are available online at https://www.mdpi.com/article/10.3390/cells12030415/s1, Figure S1: Histology of the tissues used for organ culture in the native state, Figure S2: Negative controls, prepubertal tissue, Figure S3: Mayer’s Hematoxylin staining of the tissue cultured under all 3 conditions, Figure S4: SOX9 expression in Sertoli cell nuclei, Figure S5: Expression of the BTB component CLDN11 in Sertoli cells in the native prepubertal tissue and after 3 weeks of culture, Figure S6, INSL3 expression in the native and cultured prepubertal tissue and controls, Figure S7: CYP17A1 expression in the cultured prepubertal tissue, Figure S8: Weak UTF1 expression in SOX9+ Sertoli cells in cultured tissue vs. uncultured tissue, Figure S9: DDX4/Ki-67 staining of cultured tissue from 9-year-old sickle cell disease patient, Figure S10: Atypical staining of tubular cells from cultured tissue expressing STAR, INSL3 and CYP17A1, Figure S11: Larger round mitochondria in undifferentiated spermatogonia of the 12-year-old XIAP deficiency patient, Figure S12: Adult human testicular tissue cultivated for 2 weeks in different media, Table S1: Primary antibody information, Table S2: Secondary antibody information, Table S3: Organ/organotypic culture using human prepubertal testis.
