# Supplementary material for: Germ Cell Maintenance and Sustained Testosterone and Precursor Hormone Production in Human Prepubertal Testis Organ Culture with Tissues from Boys 7 Years+ under Conditions from Adult Testicular Tissue
Source: Cells. 2023 Jan 26;12(3):415. doi: 10.3390/cells12030415 (PMC9913959; doi:10.3390/cells12030415)
Supplement: Supplementary file 1 [file cells-12-00415-s001.zip › cells-2132343-Supplementary.pdf]

Supplementary Material

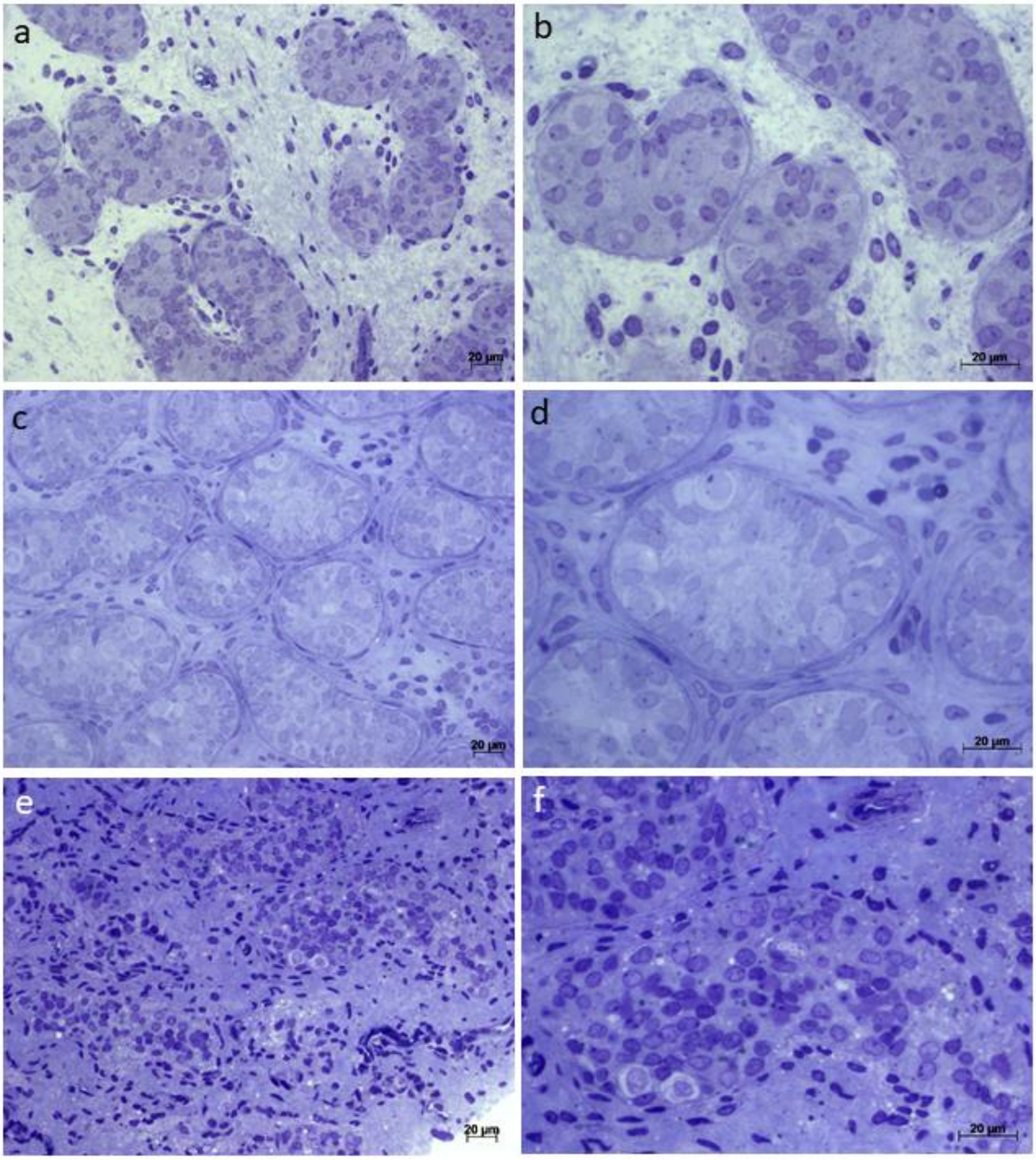

Figure S1 Histology of the tissues used for organ culture in the native state, shown on semi-thin sections stained with toluidine blue and pyronine. (**a**, **b**) 7-year-old ß-thalassemia major patient; (**c**, **d**) 12-year-old XIAP deficiency patient; (**e**, **f**) 9-year-old sickle cell disease patient.

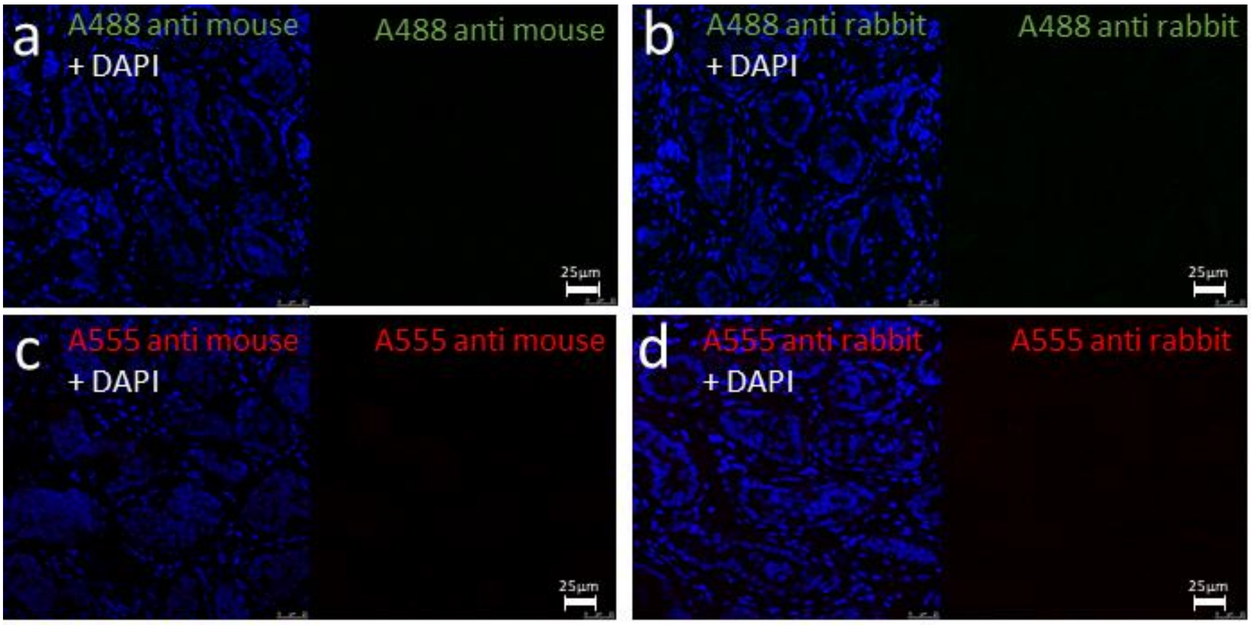

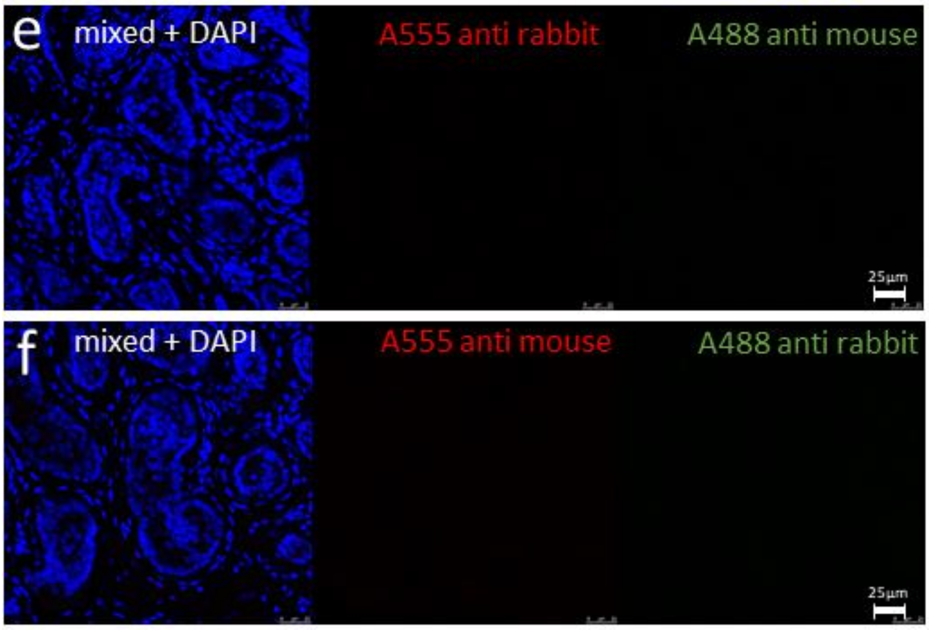

a Alexa 488 anti mouse, DAPI **b** Alexa 488 anti rabbit, DAPI **c** Alexa 555 anti mouse, DAPI **d** Alexa 555 anti rabbit, DAPI **e & f** mixed A488 A555, antibodies from contrary species.

Figure S2 Negative controls, prepubertal tissue.

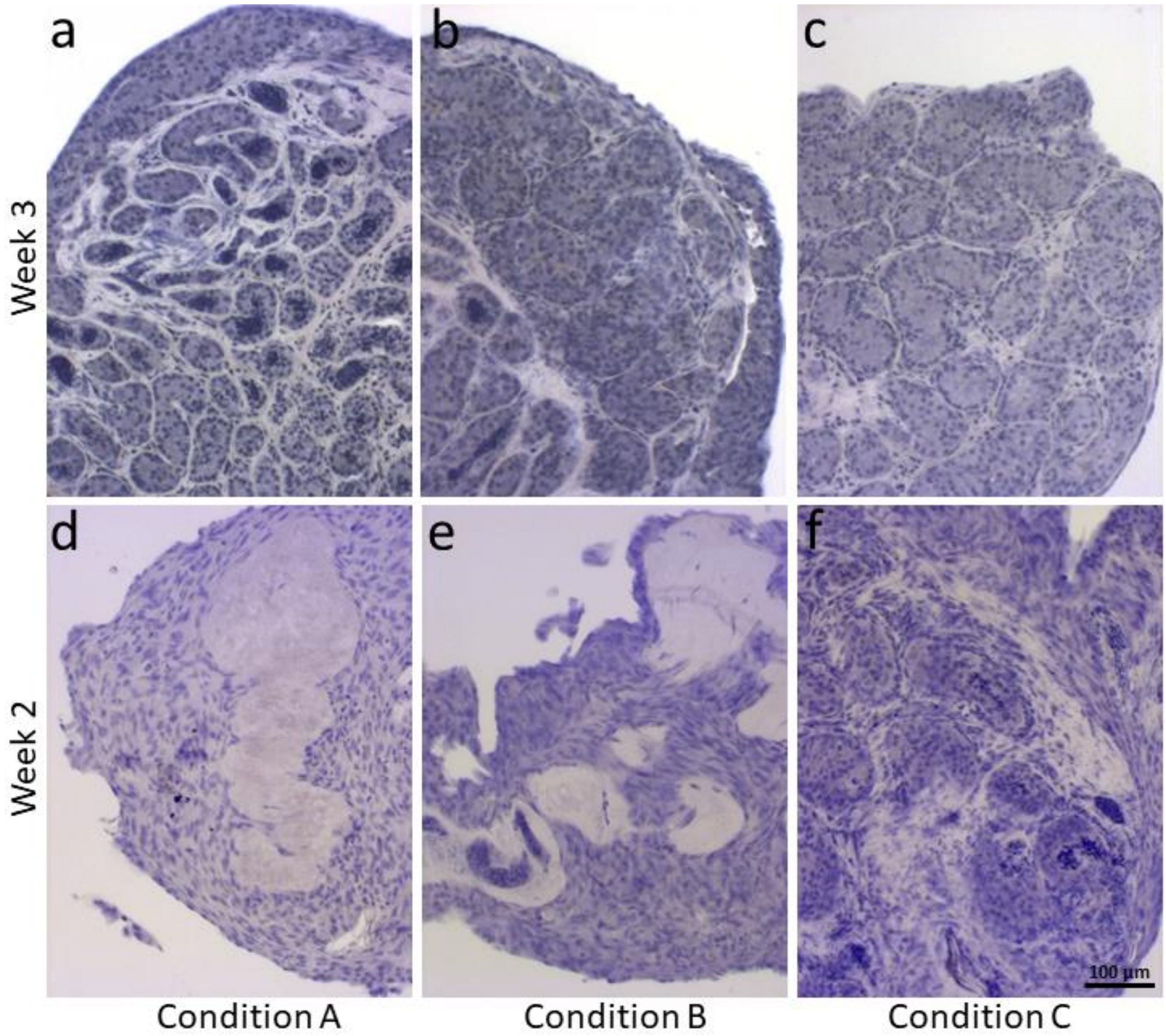

Figure S3 Mayer's Hematoxylin staining of the tissue cultured under all 3 conditions. (**a-c**) 12-year-old with XIAP deficiency, week 3. (**d-f**) 9-year-old with sickle cell disease, insufficient tissue preservation with many fibrous areas, week 2.

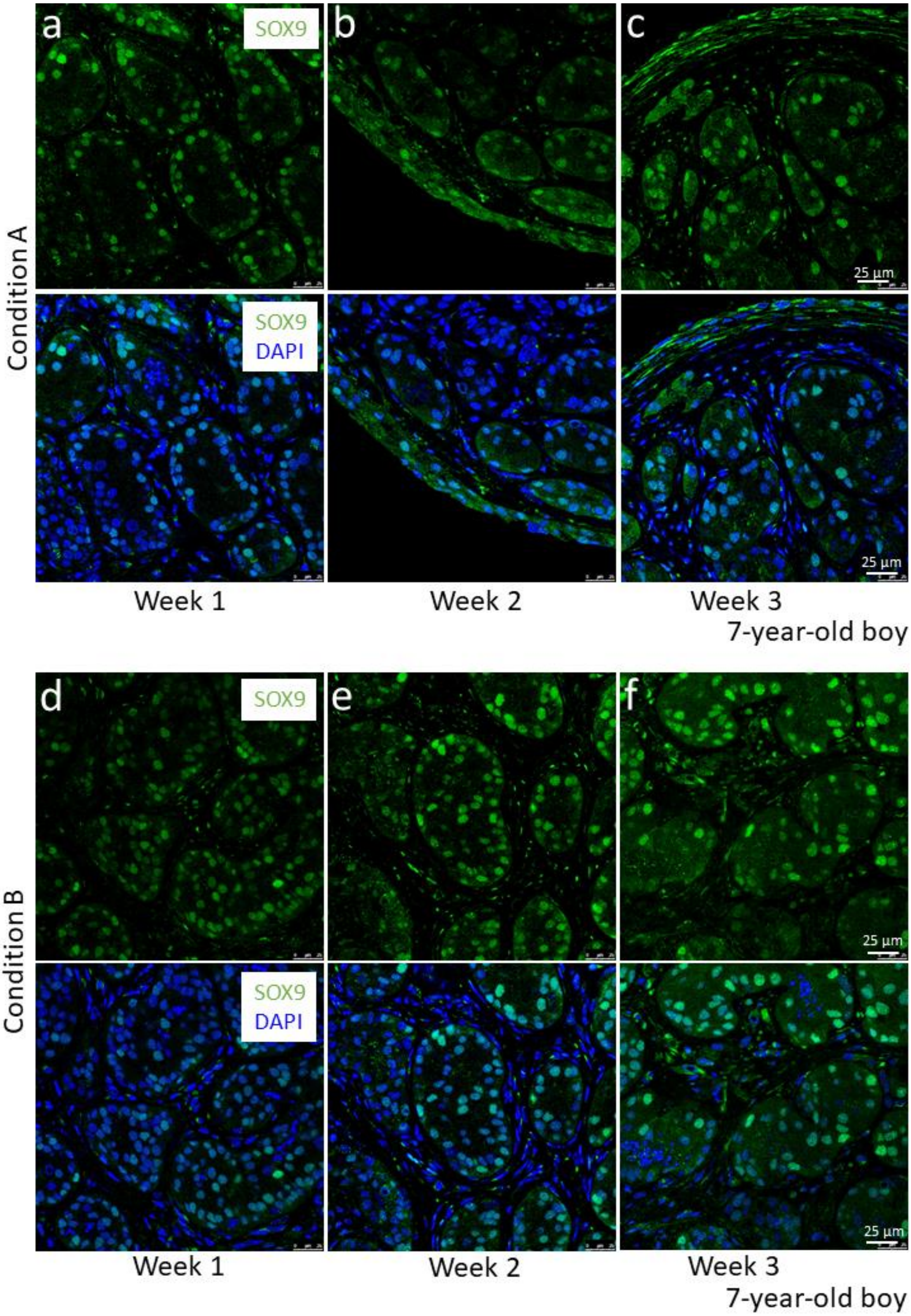

Figure S4 (1/2) SOX9 expression in Sertoli cell nuclei.

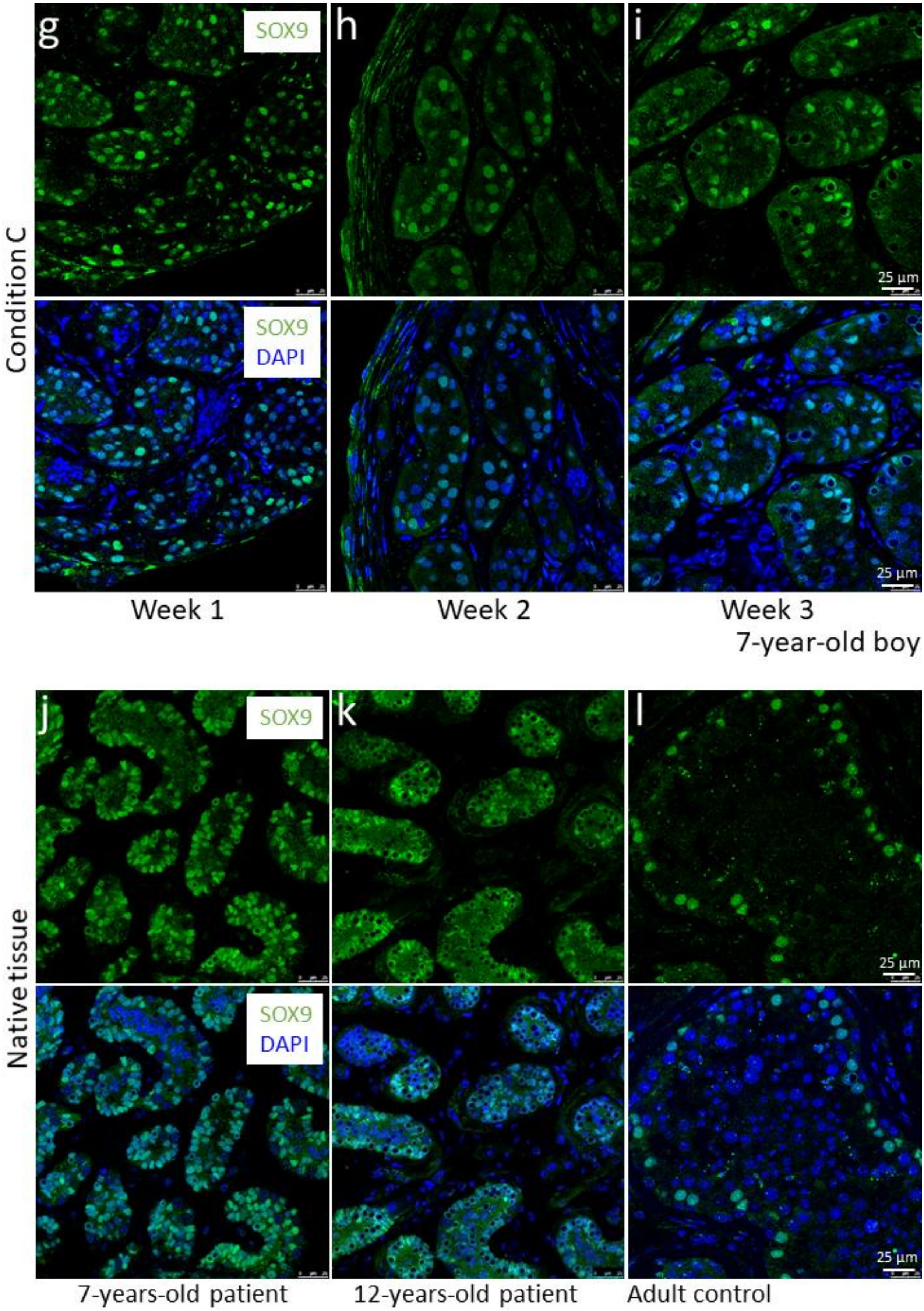

Figure S4 (2/2) SOX9 expression in Sertoli cell nuclei.

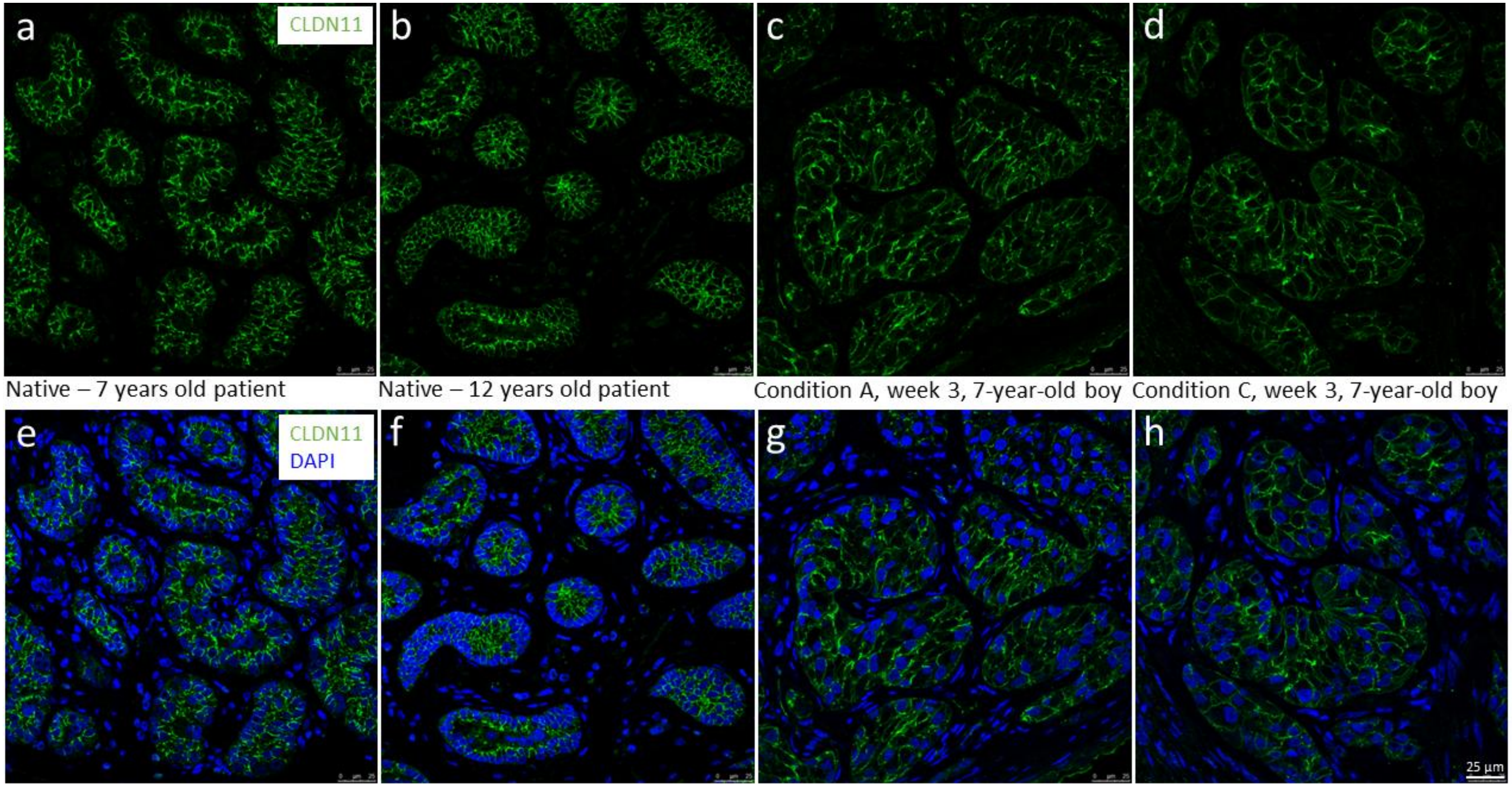

Figure S5 Expression of the BTB component CLDN11 in Sertoli cells in the native prepubertal tissue and after 3 weeks of culture. For reasons of conciseness, Condition B, which showed a similar result, is not presented here.

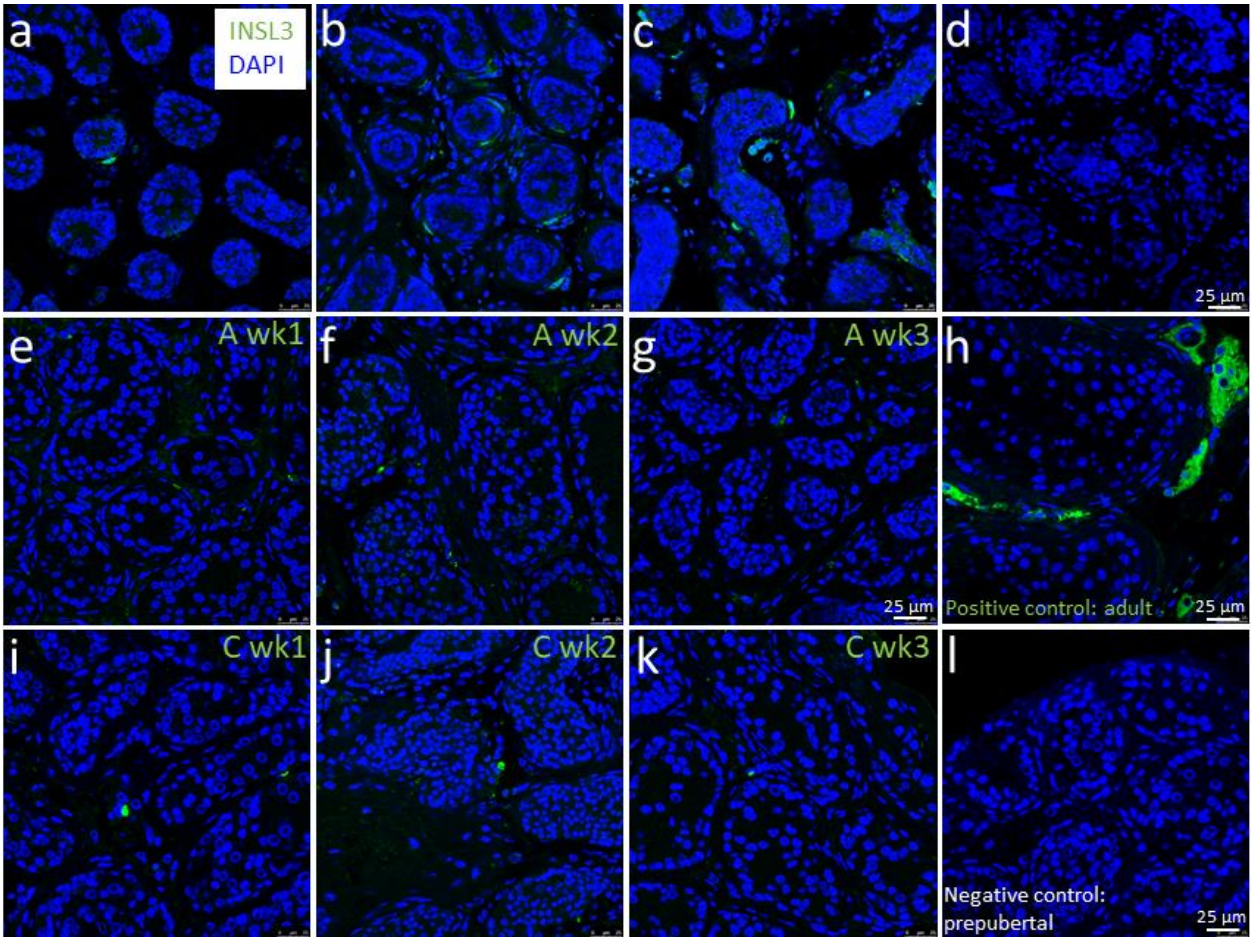

Figure S6 INSL3 expression in the native and cultured prepubertal tissue and controls. (**a-c**) Native tissue of the 12-year-old patient, and (**d**) of the 7-year-old patient. (**e, f, g, i, j, k**) Cultured tissue of the 12-year-old patient. (**h, l**) Controls.

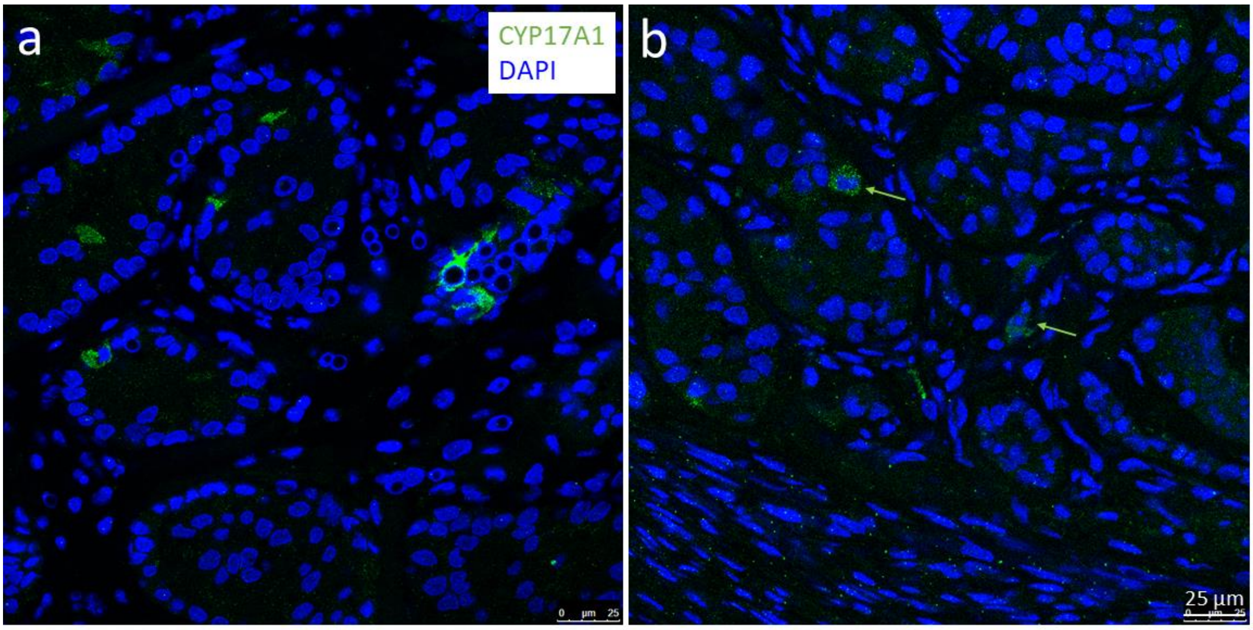

Condition C, week 3, 12-year-old patient Condition C, week 3, 7-year-old patient

Figure S7 CYP17A1 expression in the cultured prepubertal tissue.

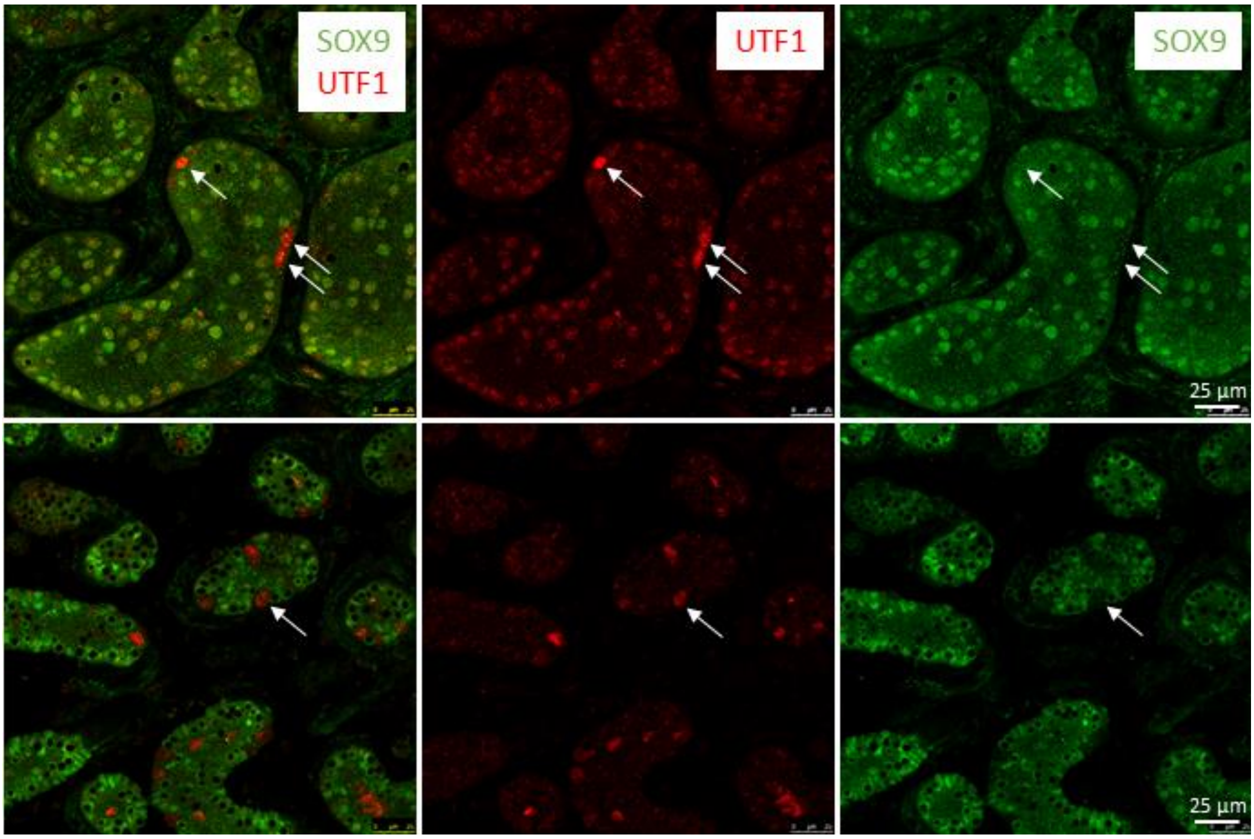

Figure S8 Weak UTF1 expression in SOX9+ Sertoli cells in cultured tissue (upper row) vs. uncultured tissue (bottom row). UTF1+ spermatogonia marked by arrows.

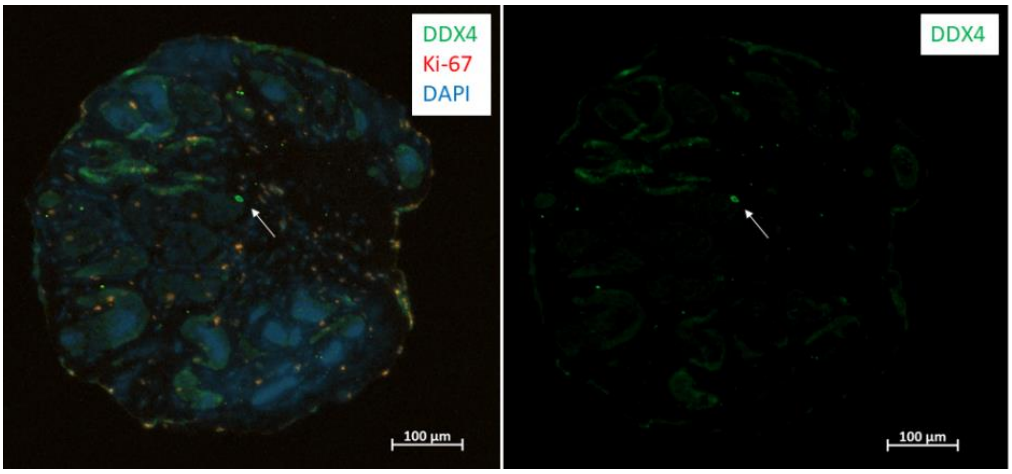

Figure S9 DDX4/Ki-67 staining of cultured tissue from 9-year-old sickle cell disease patient, condition C, week 1.

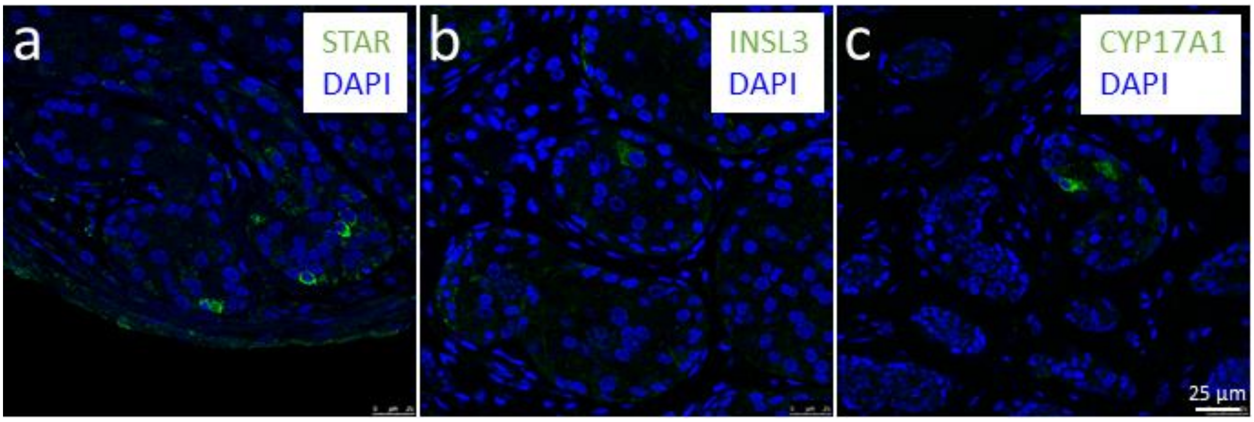

Figure S10 Atypical staining of tubular cells from cultured tissue expressing (**a**) STAR, (**b**) INSL3, (**c**) CYP17A1.

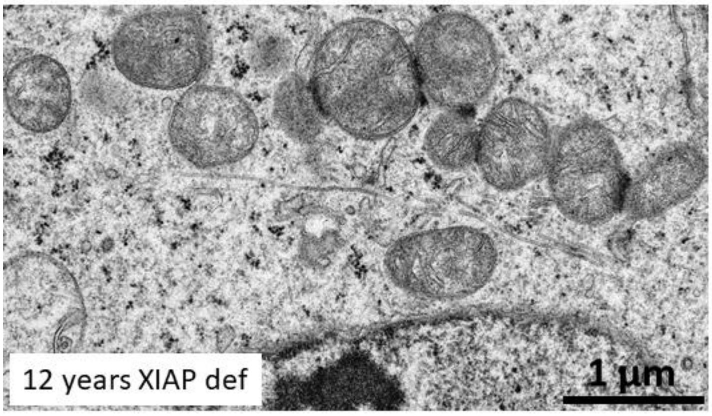

Figure S11 Larger round mitochondria in undifferentiated SPG of the 12-year-old XIAP deficiency patient.

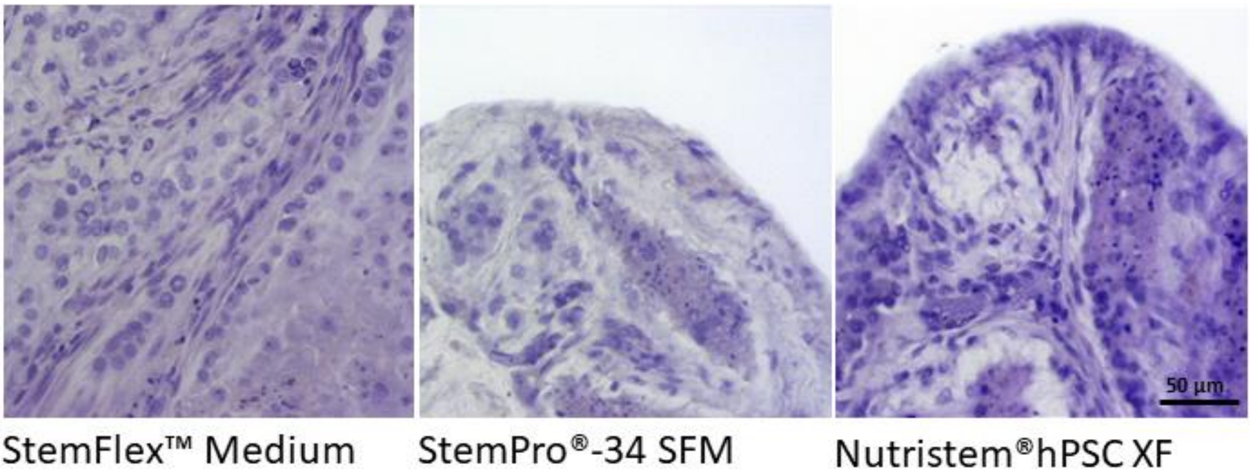

Figure S12 Adult human testicular tissue cultivated for 2 weeks in different media.

Supplementary table S1: Primary antibody information

Antigen	Company	Order number	Species	Clonality	Dilution
**BOULE**	Santa Cruz Biotechnology	sc-166660	mouse	monoclonal	1:100
**CLDN11**	Invitrogen	36-4500	rabbit	polyclonal	1:50
**CYP17A1**	Santa Cruz Biotechnology	sc-374244	mouse	monoclonal	1:500
**DDX4**	Abcam	ab13840	rabbit	polyclonal	1:500
**INSL3**	Abcam	ab227974	rabbit	polyclonal	1:100
**LAMA1**	Abcam	ab210954	mouse	monoclonal	1:20
**MKI67**	Agilent Technologies	M7240	mouse	monoclonal	1:50
**PIWIL4**	Sigma-Aldrich	HPA036588	rabbit	polyclonal	1:100
**SMA**	Sigma-Aldrich	A2547	mouse	monoclonal	1:500
**SOX9**	EMD Millipore	AB5535	rabbit	polyclonal	1:100
**STAR**	Sigma-Aldrich	HPA023644	rabbit	polyclonal	1:100
**UTF1**	EMD Millipore	MAB4337	mouse	monoclonal	1:50

Supplementary table S2: Secondary antibody information

Secondary antibody name	Company	Order number	Dilution
Alexa Fluor 488 goat anti-mouse IgG	Invitrogen	A11001	1:200
Alexa Fluor 488 goat anti-rabbit IgG	Invitrogen	A11034	1:200
Alexa Fluor 555 goat anti-mouse IgG	Life technologies	A21422	1:80
Alexa Fluor 555 goat anti-rabbit IgG	Life technologies	A21429	1:80

Supplementary table 3: Organ/organotypic culture using human prepubertal testis tissues (2017-2022)

	De Michele et al. 2017	De Michele et al. 2018	Medrano et al. 2018	Portela et al. 2019	Kurek et al. 2021	Wang et al. 2022	Aden et al. 2023
**N (experiment)**	3	5	4	9	16	4	3 (2)
**Donor tissue**	2-12 y/o	2-12 y/o	7-14 y/o	6-14 y/o	1.6-13.4 y/o	0.5-1.4 y/o	7-12 y/o
**Experiment length**	139d	139d	70d	35d	14d	60d	21d
**Germ cell markers**	MAGEA4[1]	MAGEA4[1], SYCP3[1], BOULE[1], ACE[1], PRM2[1], TNP1[1]	PLZF[2], FGFR3[2], UTF1[1, 2], SALL4[2], c-KIT[1], DDX4[1,] DAZL[2,] SYCP3[1, 2], ACR[2]	MAGEA4[1]	DDX4[1], MAGEA4[1], POU5F1[1]	ACR[1], BOULE[1], CREM[1], DDX4[1], GAGE[1], MAGEA[1], PRM1[1]	BOULE[1], DDX4[1], PIWIL4[1]
**SPG count after culture**	↓	↓	↓	↓	↓	↓	↓
**Most advanced germ cell type during culture**	not examined	SYCP3+ SPCs, BOULE+ SPCs/ rSPT, rare ACE+ eSPT	SYCP3+ cells	SPG	not examined	BOULE+ SPCs	SPG
**SPG proliferation markers**	MAGEA4/Ki-67[1]	MAGEA4/Ki-67[1]	not examined	MAGEA4/PCNA[1]	only Ki-67[1]	Morphology, Ki-67[1]	DDX4/Ki-67[1], PIWIL4/Ki-67[1]
**Proliferative SPG during culture**	proliferating SPG↓, ratio* ↑ (maintained until d64)	proliferating SPG↓, ratio* ↑ (maintained until d139)	not examined	↓	Ki-67↑	Ki-67↑	proliferation maintained
**Markers used for SC detection**	AMH[1], AR[1], GDNF[1]	AMH[1], AR[1], GDNF[1], SOX9[1]	AMH[1], AR[1], SOX9[1, 2], ZO1[1]	AMH[1], AR[1]	-	AMH[1], AR[1], SOX9[1], ZO1[1]	ultrastructure[3], SOX9[1], CLDN11[1]
**SCs during culture**	AMH ↓, GDNF, AR constant, AMH secretion↓	AMH↓ (ns), SOX9, AR, GDNF constant, AMH secretion↓	SOX9↓, AMH↓, SOX9/AR↓, ZO1 unorganized, AMH secretion↓	AMH↓, AR not expressed	not examined	SOX9↑, AMH↓, AR induced	SOX9, CLDN11 maintained
**LC markers**	3β-HSD[1]	STAR[1]	STAR[2]	-	-	CYP17A1[1], STAR[1]	Morphology, ultrastructure[3], CYP17A1[1], INSL3[1], STAR[1]
**LCs during culture**	no significant changes, testosterone secretion	STAR↑, testosterone secrection	STAR↑ in one condition after 14 days, testosterone secretion	histologically not examined, testosterone secretion	not examined	STAR induced, CYP17A1 maintained	CYP17A1↑, STAR present, INSL3 partially present, steroid hormones secreted
**PTC markers**	-	-	SMA[2]	-	LAMA1[1], LAMA5[1]; type IV collagen[1]; fibronectin[1]	SMA[1]	SMA[1], LAMA1[1]
**PTCs during culture**	not examined	not examined	SMA↑	not examined	LAMA1↓ and weak seminiferous LAMA 5 expression in some samples	induction of SMA in peritubular walls	SMA↑
**Supernatant analysis**	testosterone, AMH, LDH	testosterone, AMH	testosterone, AMH	testosterone	no	no	progesterone, 17-OHP, androstenedione, testosterone, estradiol via LCMS/MS

1 IHC 2 PCR 3 TEM *ratio = proliferating SPG/total numer of SPG

Abbreviations: **17-OHP**, 17α-hydroxyprogesterone; **3β-HSD**, 3β-hydroxysteroid dehydrogenase; **ACE**, angiotensin I converting enzyme; **ACR**, acrosin; **AMH**, anti-muellerian hormone; **AR**, androgen receptor; **BOULE** (**BOLL**), boule-like RNA binding protein; **CLDN11**, claudin 11; **c-KIT** (**KIT**), KIT proto-oncogene, receptor tyrosine kinase; **CREM**, CAMP-responsive element modulator; **CYP17A1**, cytochrome P450 family 17 subfamily A member 1; **DAZL**, deleted in azoospermia-like; **DDX4**, DEAD-box helicase 4; **FGFR3**, fibroblast growth factor receptor 3; **GAGE**, G antigen; **GDNF**, glial cell derived neurotrophic factor; **IHC**, immunohistochemistry; **INSL3**, insulin like 3; **Ki-67**, marker of proliferation Ki-67; **LAMA1**, laminin subunit alpha-1; **LAMA5**, laminin subunit alpha-5; **MAGEA**, melanoma-associated antigen A (family); **MAGEA4**, melanoma-associated antigen 4; **PCNA**, proliferating cell nuclear antigen; **PCR**, polymerase chain reaction; **PIWIL4**, piwi like RNA-mediated gene silencing 4; **PLZF** (**ZBTB16**), zinc finger and BTB domain-containing protein 16; **POU5F1**, POU class 5 homeobox 1; **PRM1**, protamine 1; **PRM2**, protamine 2; **SALL4**, spalt like transcription factor 4; **SMA** (**ACTA2**), actin alpha 2, smooth muscle; **SOX9**, SRY-box transcription factor 9; **SPCs**, spermatocytes; **SPG**, spermatogonia; **eSPTs**, elongated spermatids; **rSPTs**, round spermatids; **STAR**, steroidogenic acute regulatory protein; **SYCP3**, synaptonemal complex protein 3; **TEM**, transmission electron microscopy; **TNP1**, transition protein 1; **UTF1**, undifferentiated embryonic cell transcription factor 1; **ZO1** (**TJP1**), tight junction protein 1
